# Ubiquitin modulates 26*S* proteasome conformational dynamics and promotes substrate degradation

**DOI:** 10.1126/sciadv.add9520

**Published:** 2022-12-23

**Authors:** Erik Jonsson, Zaw Min Htet, Jared A. M. Bard, Ken C. Dong, Andreas Martin

**Affiliations:** ^1^ Department of Molecular and Cell Biology, University of California at Berkeley, Berkeley, CA 94720, USA.; ^2^ California Institute for Quantitative Biosciences, University of California at Berkeley, Berkeley, CA 94720, USA.; ^3^ Howard Hughes Medical Institute, University of California at Berkeley, Berkeley, CA 94720, USA.

## Abstract

The 26*S* proteasome recognizes thousands of appropriate protein substrates in eukaryotic cells through attached ubiquitin chains and uses its adenosine triphosphatase (ATPase) motor for mechanical unfolding and translocation into a proteolytic chamber. Here, we used single-molecule Förster resonance energy transfer measurements to monitor the conformational dynamics of the proteasome, observe individual substrates during their progression toward degradation, and elucidate how these processes are regulated by ubiquitin chains. Rapid transitions between engagement- and processing-competent proteasome conformations control substrate access to the ATPase motor. Ubiquitin chain binding functions as an allosteric regulator to slow these transitions, stabilize the engagement-competent state, and aid substrate capture to accelerate degradation initiation. Upon substrate engagement, the proteasome remains in processing-competent states for translocation and unfolding, except for apparent motor slips when encountering stably folded domains. Our studies revealed how ubiquitin chains allosterically regulate degradation initiation, which ensures substrate selectivity in a crowded cellular environment.

## Teaser

Ubiquitin chains allosterically stabilize the engagement-competent 26S proteasome conformation and promote substate capture.

## INTRODUCTION

Eukaryotic cell viability critically depends on the 26*S* proteasome, which is responsible for protein homeostasis, quality control, and the degradation of numerous regulatory proteins (*1*, *2*). This major protease of the AAA^+^ [adenosine triphosphatases (ATPases) associated with diverse cellular activities] family recognizes protein substrates that have been modified with lysine-attached polyubiquitin chains, deubiquitinates them, and uses its ATPase motor for mechanical unfolding and translocation into an internal chamber for proteolytic cleavage (*3*). To degrade thousands of diverse proteins in a controlled manner, the 26*S* proteasome needs to combine rapid substrate processing and high promiscuity with sufficient selectivity to avoid unregulated proteolysis of cellular proteins in general. This regulation is accomplished by the intricate architecture of the proteasome and its major conformational changes that help coordinate individual degradation steps (*4*), as well as the bipartite character of a substrate’s degradation signal, which consists of a polyubiquitin modification for targeting to the proteasome and an unstructured initiation region of at least 20 to 25 residues for engagement by the proteasomal ATPase motor (*4*–*10*).

Substrate cleavage by the proteasome occurs in the internal degradation chamber of the barrel-shaped 20*S* core peptidase, with access through axial gates being controlled by the 19*S* regulatory particle (*11*–*13*). This regulatory particle consists of the base and lid subcomplexes and functions in ubiquitin-mediated substrate recognition, deubiquitination, adenosine 5′-triphosphate (ATP)–dependent mechanical unfolding, and translocation into the 20*S* core. The base subcomplex contains 10 subunits, including three ubiquitin receptors, regulatory particle non-ATPase 1 (Rpn1)(*14*), Rpn10 (*15*), and Rpn13 (*16*), and a heterohexameric ATPase motor formed by six distinct subunits, regulatory particle triphosphatase 1-6 (Rpt1 to Rpt6), with an N-terminal domain ring (N-ring) stacked on top of an AAA domain ring (*14*, *17*–*19*). After ubiquitin binding to a receptor, the flexible initiation region of the substrate must reach through the N-ring and into the ATPase ring to engage with pore loops that transduce ATP hydrolysis into mechanical pulling for substrate unfolding and translocation into the 20*S* core (*4*, *20*, *21*). The nine-subunit lid subcomplex is bound to one side of the base and contains the deubiquitinating enzyme (DUB) Rpn11 (*22*–*26*), which catalyzes the cotranslocational deubiquitination of substrates before their entry into the AAA^+^ motor (*27*).

**Fig. 1.**
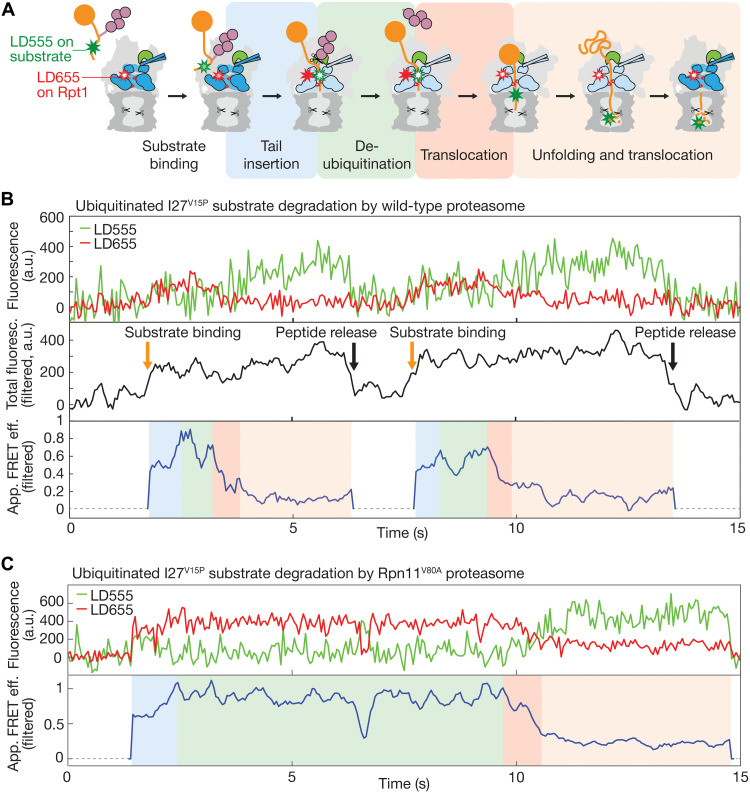
Direct observation of substrate processing. (**A**) Cartoon representation depicting the sequential stages for the proteasomal degradation of a ubiquitinated substrate. The 20*S* core peptidase is shown in dark gray, and the 19*S* regulatory particle is shown in light gray, with Rpn11 highlighted in green, the Rpt1 to Rpt6 ATPase ring highlighted in dark blue for the s1 conformational state and light blue for non-s1 states, and pore 1 loops shown as red hooks. The substrate is depicted in orange with the ubiquitin chain in purple. Green and red stars represent the substrate-attached LD555 donor and proteasome-attached LD655 acceptor dyes, respectively, with the filling indicating their expected fluorescence upon Foerster resonance energy transfer (FRET) during the different stages of substrate processing (see also fig. S1B). (**B**) Representative traces showing two consecutive events of ubiquitinated titin I27^V15P^ degradation by a single immobilized wild-type proteasome excited with a 532-nm laser, with the donor fluorescence signal in green and the acceptor fluorescence shown in red at the top, the total fluorescence (filtered sum of donor and acceptor fluorescence) shown in black in the middle, and the filtered apparent FRET efficiency shown in blue at the bottom. The total fluorescence signal with a threshold twofold above background was used to detect individual degradation events, starting with the arrival of a donor-labeled substrate (orange arrow) and ending with the release of the donor-labeled peptide product (black arrow). Outside of these degradation events the apparent FRET efficiency was not calculated and represented by a dashed line. During degradation, the fluorescence signals and apparent FRET efficiency show four phases that are shaded in different colors according to the substrate-processing steps shown in (A). (**C**) Representative fluorescence and FRET efficiency traces for the degradation of ubiquitinated I27^V15P^ substrate by partially deubiquitination-inhibited Rpn11^V80A^ mutant proteasome, which leads to an extended high–FRET efficiency phase (green shading). a.u., arbitrary units.

Previous structural studies identified multiple proteasome conformations that differ in the relative orientation and interactions of the lid, base, and core subcomplexes (*28*–*36*) and provide a global framework for the mechanism of the 26*S* proteasome (Fig. 1A). Dominant in the absence of substrate is the engagement-competent s1 state, in which the ATPase ring is not coaxially aligned with the core peptidase and Rpn11 is offset from the central processing channel, allowing substrate access to the entrance of the AAA^+^ motor. In the presence of substrate or the nonhydrolyzable ATP analog ATPγS [adenosine 5′-*O*-(3-thiotriphosphate)], the proteasome adopts various processing states named s2 to s6, or collectively non-s1 (*4*, *28*, *30*, *32*, *33*, *35*, *36*), in which the N-ring, ATPase ring, and core peptidase are coaxially aligned to form a continuous channel for efficient substrate translocation. Furthermore, Rpn11 is moved to a centrally aligned position that partially obstructs the motor entrance and thus facilitates en bloc ubiquitin chain removal from substrates as they are translocated into the motor (Fig. 1A) (*27*, *35*, *36*). Structures of substrate-bound proteasomes in non-s1 states show four or five Rpt subunits with their pore loops contacting the substrate polypeptide and adopting different spiral staircase arrangements around the hexameric ring (*35*, *36*), suggesting that translocation occurs by a hand-over-hand mechanism with a basic step size of two amino acids per hydrolyzed ATP, similar to related AAA^+^ protein translocases (*37*–*39*).

Previous ensemble Foerster resonance energy transfer (FRET)–based measurements revealed that the major conformational changes from the s1 to non-s1 states are triggered by insertion and motor engagement of the substrate’s flexible initiation region (Fig. 1A) (*4*) and thus seem to play an important role in coordinating subsequent substrate-processing steps, including translocation-coupled deubiquitination. Interactions at the lid-base interface, particularly between the lid subunit Rpn5 and the Rpt3 ATPase, were found to stabilize the s1 state, and their disruption causes inhibited degradation due to compromised substrate engagement (*40*). Furthermore, the nucleotide state and hydrolysis activity of individual ATPase subunits, for instance, Rpt4 and Rpt6, affect the proteasome’s conformational equilibrium (*40*, *41*).

Despite these previous insights, the detailed mechanisms underlying substrate engagement, deubiquitination, mechanical unfolding, and translocation, as well as the role of proteasome conformational transitions for the individual processing steps remain largely elusive. Furthermore, ubiquitin modifications were observed to facilitate substrate degradation (*42*, *43*), yet their effects beyond substrate recruitment, for instance, on degradation initiation, mechanical processing, or the proteasome conformational states are unknown.

Here, we used single-molecule FRET measurements to monitor the conformational dynamics of the proteasome and observe individual substrate molecules on their way through the regulatory particle toward degradation. These experiments revealed the velocity of translocation, and translocation-coupled deubiquitination, and the rapid conformational switching of the proteasome regulatory particle that depends on a network of interactions at the lid-base interface and plays an important role in selective substrate engagement. Furthermore, we uncovered how ubiquitin chain binding to the proteasome allosterically affects these conformational transitions and facilitates degradation initiation, providing insights into the mechanism and regulation of ubiquitin-dependent substrate degradation by the proteasome.

## RESULTS

### Substrate progression through the 26*S* proteasome

**Fig. 2.**
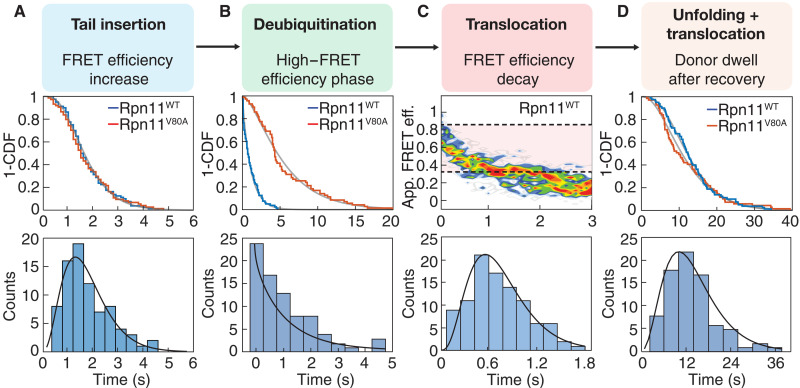
Analyses of processing phases during the degradation of ubiquitinated I27^V15P^ substrate. (**A**) Top: 1-CDF, 1–cumulative distribution function, or survival plots for the FRET efficiency increase phases during substrate degradation by wild-type (blue, *N* = 80) and Rpn11^V80A^ mutant (red, *N* = 74) proteasomes. Fit lines in gray are based on the cumulative distribution of the gamma function derived from fitting the dwell time distributions (*P* = 0.6197, Mantel-Cox test). Bottom: Histogram for the duration of the FRET efficiency increase phase during degradation by wild-type proteasome, with the fit to a gamma distribution shown as a gray line. (**B**) Top: Survival plots for the high–FRET efficiency phase during substrate degradation by wild-type (blue, *N* = 80) and Rpn11^V80A^ mutant (red, *N* = 71) proteasomes (*P* < 0.0001, Mantel-Cox test). Bottom: Histogram for the duration of the high–FRET efficiency phase during degradation by wild-type proteasome, with the fit to a gamma distribution shown as a gray line. (**C**) Top: Contour plot for multiple trajectories of FRET efficiency decay during substrate degradation by wild-type proteasome. Trajectories were synchronized using the point where the decay from the high–FRET efficiency value initiates (*N* = 98). The duration of the apparent FRET efficiency change from 0.82 to 0.32 (shaded in pink) was used to determine translocation velocity. Bottom: Histogram for the duration of the apparent FRET efficiency decay from 0.82 to 0.32 during substrate degradation by wild-type proteasome, with the fit to a gamma distribution shown as a gray line. (**D**) Top: Survival plots for the dwell times of donor dyes after fluorescence recovery during substrate degradation by wild-type (blue, *N* = 80) and Rpn11^V80A^ mutant (red, *N* = 69) proteasomes (*P* = 0.6891, Mantel-Cox test). Bottom: Histogram for the duration of the donor fluorescence dwell after recovery during degradation by wild-type proteasome, with the fit to a gamma distribution shown as a gray line.

We used single-molecule FRET measurements to observe individual substrates as they translocate through the proteasome regulatory particle on their way to degradation. Our model substrates consisted of a titin I27 domain, either wild type or carrying the destabilizing mutations V13P and V15P (*4*, *44*), and a C-terminal cyclin B–derived unstructured initiation region that was labeled with a LD555 or Cy3 donor dye on an engineered C127 and ubiquitinated on a single lysine K117 (fig. S1, A and B). Reconstituted proteasomes with a LD655 acceptor dye–labeled azido-phenylalanine (AzF) in the linker between the N domain and the AAA^+^ domain of the Rpt1 ATPase subunit (I191AzF; fig. S1A) were immobilized to the surface of a microscope coverslip, substrates were added to the reaction chamber, and fluorescence signals were detected in a total internal reflection fluorescence (TIRF) microscope (fig. S2). By monitoring variations in the fluorescence intensities of the donor and acceptor dyes, we could observe individual substrate molecules as they enter the proteasome and progress through the central channel during the various stages of processing (Fig. 1A and fig. S2D). Proteasomes often showed several sequential degradation events, and a representative trace with two consecutive degradations of ubiquitinated titin I27^V15P^ by a single proteasome is depicted in Fig. 1B. All degradation events exhibit an evolution of the apparent FRET efficiency, starting from an intermediate level, followed by an increase and a short dwell in a high-FRET state, before gradually decaying to a background level. The decay in apparent FRET efficiency occurs with a concomitant increase in donor fluorescence that persists for a while before disappearing in a single step, which confirms the observation of a single-labeled substrate molecule. These apparent FRET efficiency and fluorescence profiles are consistent with a substrate-attached donor dye localizing near the regulatory particle upon substrate binding; entering the central processing channel of the ATPase motor; translocating toward, past, and away from the motor-attached acceptor dye; and then progressing into the 20*S* core particle, where substrate cleavage and diffusion of the donor-labeled peptide out of the proteolytic chamber lead to disappearance of the donor signal (Fig. 1, A and B). The expected anticorrelated behavior of donor and acceptor fluorescence signals is in part obscured by the noise from other labeled substrate molecules in solution, and we use the total fluorescence, i.e., the sum of donor and acceptor signals, to reliably detect substrate-processing events (Fig. 1B). Measuring the signal increase from the start of the reaction to the point of highest apparent FRET efficiency gave a time constant of τ_ins_ = 1.8 ± 0.1 s (*N* = 80; Fig. 2A), which is in excellent agreement with previous bulk measurements of substrate tail insertion (*4*) and indicates full activity of the surface-immobilized proteasomes. This tail insertion includes the passive diffusion of the substrate’s C-terminal initiation region into the central channel, the engagement by the ATPase motor, and the onset of translocation. On the basis of our structure of the substrate-engaged proteasome (*35*), we placed the donor dye on the substrate’s unstructured tail such that it localizes between the N-ring and ATPase ring and thereby causes a high-FRET signal when the tail is fully inserted and the substrate-linked ubiquitin chain reaches the Rpn11 deubiquitinase above the N-ring (fig. S1B). Deubiquitination is therefore expected to occur during the high–FRET efficiency dwell, which we measured to last only for τ_DUB_ = 1.1 ± 0.2 s (*N* = 80; Fig. 2B and fig. S3A). We can conclude that degradation-coupled ubiquitin chain removal proceeds at least four times faster than the 4.6 s that we previously determined in bulk measurements with a FRET-based deubiquitination assay that did not allow a separation from tail insertion or translocation immediately after deubiquitination (*4*). To verify this assignment of deubiquitination during the high-FRET phase, we introduced the Rpn11 V80A point mutation that was previously shown in bulk measurements to slow down substrate deubiquitination by ~4-fold (*27*). Consistently, we observed extended high–FRET efficiency dwells of τ_DUB_ = 5.5 ± 0.5 s (*N* = 71; Figs. 1C and 2B), whereas other phases of substrate processing, such as tail insertion, remained unaffected (Fig. 2A and table S1).

After ubiquitin chain removal, the proteasomal ATPase motor is expected to freely translocate the 24 residues between the deubiquitinated lysine K117 and the titin I27 domain in our model substrate (fig. S1B), leading to a gradual decay of the apparent FRET efficiency before the folded domain reaches the narrow entrance to the N-ring (Fig. 2C). However, the observed FRET efficiency changes cannot be used to simply calculate changes in donor-acceptor distance and translocation velocity. Although the sum of donor and acceptor fluorescence signals during substrate degradation did not show any considerable variations that could indicate protein-induced fluorescence enhancement (fig. S4C), pulling the substrate-attached donor dye through the narrow motor channel involves steric interaction with pore loops, changes the environment of the dye, and likely affects its rotatability, fluorescence anisotropy, and, consequently, FRET efficiency. To correlate the decrease in apparent FRET efficiency with substrate translocation, we therefore used a translocation reference based on a series of stalled substrate variants, in which the donor-labeled cysteine was moved from its original position C127 further toward the C terminus by increments of up to 24 amino acids. By using deubiquitination-inhibited proteasomes that contain the catalytically dead Rpn11^AXA^ mutant and adding ATPγS after substrate engagement (see Materials and Methods), we were able to specifically stall these substrate variants as previously observed in our substrate-engaged proteasome structures (*35*) and thereby place the donor dye in different positions along the processing channel of the ATPase motor. For these stalled substrate variants, we detected an overall stepwise decrease in apparent FRET efficiency, from a maximum value of ~0.82, when the donor dye is at the top of the ATPase spiral staircase, to a value of ~0.32, when the dye is moved by 24 residues to the bottom of the spiral near the gate to the 20*S* core peptidase (fig. S4A). For the degradation of our model substrates, it is expected that deubiquitination occurs during the high–FRET efficiency phase and the subsequent translocation of the 24-residue linker between K117 and the titin-folded domain (L93) leads to the decay in apparent FRET efficiency. Consistent with this substrate geometry, the temporally aligned apparent FRET efficiency traces for I27^V15P^ substrate degradation events showed an initial rapid decay from ~0.82 to ~0.32, followed by a slower decline with broader distribution that may indicate the titin-folded domain approaching the entrance to the motor channel and interfering with a continuous free translocation. Analyzing the distribution of times required for the apparent FRET efficiency decay from 0.82 to 0.32 revealed a time constant of 0.74 ± 0.1 s for the unobstructed translocation of I27^V15P^ after deubiquitination and before unfolding (Fig. 2C and table S2). Very similar times were observed for the processing of the more labile I27^V13P/V15P^ substrate by wild-type proteasomes and the I27^V15P^ substrate by Rpn11^V80A^ mutant proteasomes (0.80 ± 0.05 and 0.80 ± 0.04 s; figs. S3 and S4B and table S2), as expected if the translocation velocity before titin unfolding is not affected by the substrate’s thermodynamic stability or the kinetics of ubiquitin chain removal. These times for the apparent FRET efficiency decay during movement of ~24 residues through the central channel correspond to an approximate translocation velocity of *k*_trans_ ~ 30 amino acids s^−1^. The proteasome thus appears to thread a polypeptide substrate an order of magnitude faster than the ~2 to 3 amino acids s^−1^ suggested by a structure-derived step size of 2 amino acids ATP^−1^ (*35*) and a bulk ATP hydrolysis activity of 1.2 ATP s^−1^ (*40*). Either bulk ATPase measurements do not accurately reflect the proteasome’s hydrolysis rate during unobstructed substrate translocation or AAA^+^ motors take larger steps than implied by their static substrate-bound structures (*35*, *37*–*39*). Much larger steps of 1 to 4 nm, equivalent to 5 to 20 amino acids, were previously observed in single-molecule optical tweezer experiments with the related bacterial protease ClpXP (*45*–*48*).

By the time the motor attempts to mechanically unfold the titin domain, the donor dye has proceeded through the ATPase ring to a range within the 20*S* core particle that is no longer amenable to efficient energy transfer. Nevertheless, the prolonged persistence of the donor fluorescence at the position of the immobilized proteasome in our TIRF measurements reports on the approximate time required for substrate unfolding and translocation to the peptidase active sites, cleavage, and release of the peptides from the proteolytic chamber. We detected a donor fluorescence dwell of 13.3 ± 0.8 s for this complete processing of the I27^V15P^ substrate (Fig. 2D). Rpn11^V80A^ mutant proteasomes show a similar donor fluorescence dwell (Fig. 2F), confirming that the Rpn11 activity has no effect on the processing steps after deubiquitination.

### Observing the proteasome conformational dynamics

**Fig. 3.**
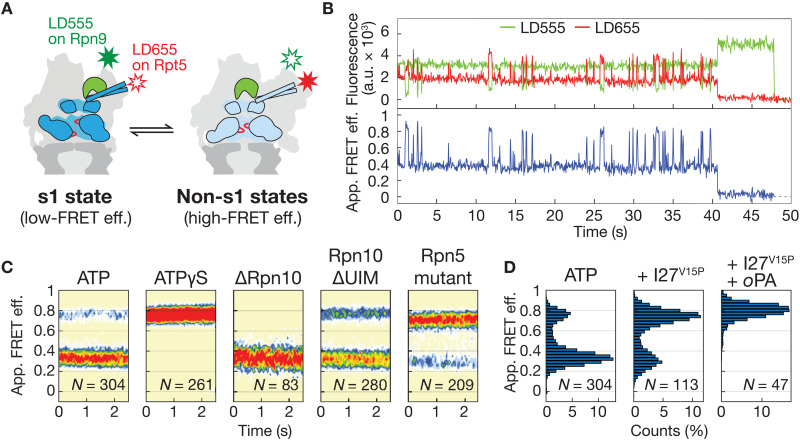
Conformational dynamics of the proteasome. (**A**) Cartoon representation for the conformational transition between the engagement-competent s1 and the processing-competent non-s1 states. A lid-attached donor dye (LD555 on Rpn9, green star) and base-attached acceptor dye (LD655 on Rpt5, red star) allow monitoring of these transitions by FRET in a conformational change assay (low apparent FRET efficiency in s1 and high apparent FRET efficiency in non-s1). (**B**) Representative trace for a wild-type proteasome in ATP excited with a 532-nm laser exhibits dynamic switching between low-FRET and high-FRET states, with raw donor fluorescence signal shown in green, the raw acceptor fluorescence signal in red, and the calculated apparent FRET efficiency in blue. (**C**) Contour plots generated from time-binned histograms of multiple traces indicate the relative occupancies of low-FRET and high-FRET states for the 26*S* proteasome in the presence of ATP and the nonhydrolyzable ATPγS, upon deletion of the entire Rpn10 or its ubiquitin-interacting motif (UIM), and upon insertion of s1-state destabilizing mutations in Rpn5. (**D**) Apparent FRET efficiency distribution of wild-type proteasome in the presence of ATP (left), upon addition of 400 nM ubiquitinated titin I27^V15P^ substrate (middle), and addition of substrate and the Rpn11 inhibitor 1,10-phenanthroline (*o*PA) at 3 mM (right).

Numerous cryo–electron microscopy (cryo-EM) studies indicated that upon substrate engagement by the AAA^+^ motor, the proteasome transitions from an engagement-competent s1 state with an accessible motor entrance to non-s1 or processing-competent states, in which Rpn11 is positioned right above the central channel for efficient cotranslational deubiquitination (*28*, *32*, *33*, *35*, *36*). During this global conformational switch, the distance between the lid subunit Rpn9 and the N-terminal coiled coil of the Rpt4/Rpt5 ATPase pair decreases by ~30 Å. We previously developed a FRET-based assay with dyes attached in those positions to monitor the proteasome conformational change upon substrate engagement in bulk measurements (*4*). Here, to investigate the conformational dynamics of individual proteasomes, we optimized this FRET system for single-molecule experiments with a LD555 donor dye attached to azidophenylalanine incorporated at position 2 of Rpn9 and a LD655 acceptor dye attached to azidophenylalanine at position 49 of Rpt5 (Fig. 3A and fig. S1A). By selecting particles with donor and acceptor signals and appropriate fluorescence intensities, we ensured that only fully assembled and singly capped proteasomes containing the lid, base, and core subcomplexes were analyzed (fig. S2C).

A representative trace for the substrate-free proteasome in the presence of ATP is depicted in Fig. 3B, indicating a dynamic switching between two conformational states (fig. S5A). Based on the positioning of the dyes, we assign the low-FRET state to s1 and the high-FRET state to non-s1 conformations (Fig. 3A). Previous structural studies revealed that the individual non-s1 states differ in their spiral staircase arrangements of the six ATPase subunits, but exhibit no global changes in the relative orientation of the lid and base subcomplexes (*32*, *35*, *36*). We therefore did not expect the FRET probes to distinguish between individual non-s1 states, and our measurements are consistent with a two-state system (fig. S5B). The histogram for the apparent FRET efficiency shows that the low-FRET s1 state is predominant, whereas the high-FRET non-s1 states are only transiently visited under these conditions (Fig. 3C). The dwell times in each state could be determined through hidden Markov modeling and allowed us to derive the kinetics for conformational switching, with *k*_s1_ = 1.2 ± 0.1 s^−1^ and *k*_non-s1_ = 4.5 ± 0.1 s^−1^ for the s1–to–non-s1 and non-s1–to–s1 transitions, respectively (fig. S5, C and D). Addition of the nonhydrolyzable ATP analog, ATPγS, shifts the histogram completely to the high-FRET non-s1 states (Fig. 3C and fig. S7A), which is in agreement with previous cryo-EM studies (*29*) as well as bulk measurements (*4*) and confirms our conformational assignment of apparent FRET efficiency levels.

Low ATP concentrations bias the conformational equilibrium toward non-s1 states, and the absence of nucleotide leads to an almost complete shift (fig. S6, A and B), similar to the scenario observed in the presence of the nonhydrolyzable ATPγS. This finding suggests that establishing all Rpt subunits in identical nucleotide states, i.e., empty or ATPγS bound, favors non-s1 conformations, whereas the presence of various nucleotide states in the Rpt hexamer during active ATP hydrolysis may provide strains that stabilize the more distorted s1 state, at least in the absence of substrate. The presence of only adenosine 5′-diphosphate, however, destabilizes the proteasome holoenzyme and leads to the dissociation of the regulatory particle from the immobilized 20*S* core, as indicated by the lack of colocalized lid and base subcomplexes in our conformational change assay (fig. S6C).

We also analyzed proteasomes containing mutations in the VTENKIF motif of the lid subunit Rpn5 that forms s1 state–specific contacts with the ATPase subunit Rpt3. Disruption of these contacts was previously found to inhibit substrate degradation by interfering with initiation (*40*). We observed that in the presence of ATP, the Rpn5 mutant proteasomes not only exhibit a bias away from the s1 state (Fig. 3C), but also switch more rapidly between conformations (*k*_s1_ = 2.5 ± 0.1 s^−1^ and *k*_non-s1_ = 4.2 ± 0.1 s^−1^; fig. S7D and table S3). These results are consistent with a mechanism of degradation inhibition by which accelerated conformational transitions and/or a shift in the equilibrium toward non-s1 states with an Rpn11-obstructed motor entrance hinder substrate engagement (*40*). This behavior of the Rpn5 mutant highlights the importance of the engagement-competent s1 state being long-lived enough to allow substrate insertion into the central channel, and it emphasizes the critical role of lid-base interactions in controlling the dynamics of the proteasome during various stages of degradation.

Holoenzymes reconstituted in the absence of the ubiquitin receptor Rpn10, which bridges the lid and base subcomplexes, show the conformational equilibrium shifted toward a low-FRET state that, according to previous EM studies, likely reflects the s1 conformation (Fig. 3C and fig. S7B) (*49*). The apparent FRET efficiency values observed for the non-s1 states of ΔRpn10 proteasomes are lower compared to wild-type proteasomes, yet we assume that they represent functional, processing-competent conformations with possibly reoriented dyes, as we measured a robust, albeit lower substrate degradation activity that produced peptides similar to those of wild-type proteasomes (fig. S9). Consistent with these effects in ATP, ΔRpn10 proteasomes with bound ATPγS still show a bimodal distribution of the apparent FRET efficiency histogram and a considerable population of s1 state particles (fig. S8), suggesting that Rpn10 plays a role in stabilizing non-s1 states, at least in the absence of substrate. This non-s1 stabilization appears to be contributed by Rpn10’s globular (VWA) domain, as deletion of Rpn10’s C-terminal ubiquitin-interacting motif (UIM) alone shifts the equilibrium compared to wild-type proteasomes toward non-s1 states (Fig. 3C and fig. S7C), and it is consistent with Rpn10’s previously described role in stabilizing the lid-base assembly (*50*, *51*). Together, our data indicate the presence of an intricate system of interactions at the lid-base interface that differentially stabilize the s1 and non-s1 states and thus allow for dynamic conformational transitions and equilibrium shifts depending on the stage of substrate processing.

### Conformational response to substrate engagement and processing

**Fig. 4.**
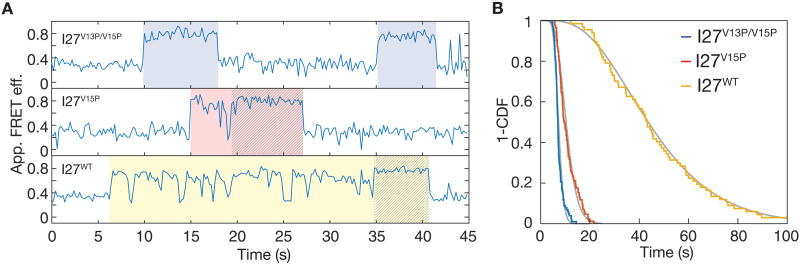
Proteasome conformational changes during substrate degradation. (**A**) Representative traces of the apparent FRET efficiency for the conformational dynamics of the proteasome during SspB-mediated degradation of the I27^V13P/V15P^, I27^V15P^, and I27^WT^ substrates with different thermodynamic stabilities (traces for I27^V13P/V15P^ and I27^V15P^ were down-sampled to 5 Hz to match I27^WT^; for non–down-sampled traces, see fig. S11). Substrate-processing dwells are highlighted by shading in blue (I27^V13P/V15P^), red (I27^V15P^), and yellow (I27^WT^), and their lengths and the frequency of excursion to the low-FRET s1 state increase with increasing substrate stability. The last 3 to 5 s of traces show an excursion-free high-FRET state that is indicated by hatching. (**B**) Survival plots for the substrate-processing dwells during SspB-mediated degradation of the I27^V13P/V15P^ (blue, *N* = 102), I27^V15P^ (red, *N* = 85), and I27^WT^ (yellow, *N* = 67) substrate variants. Fit lines in gray are based on the cumulative distribution of the gamma function derived from fitting the dwell time distributions. The survival plots were compared using the Mantel-Cox test, yielding *P* < 0.0001 between each type of substrate.

The addition of ubiquitinated substrates at saturating concentrations shifts the proteasome conformational equilibrium toward non-s1 states (Fig. 3D), in agreement with previous findings that substrate engagement induces an s1–to–non-s1 transition (*4*, *28*). The minor s1 peak in the histogram is explained by the asynchronicity of individual particles and the fact that proteasomes appear to “idle” in the s1 state between degradation events. Consistently, the conformational equilibrium could be fully shifted to high-FRET states by stalling substrate degradation at the deubiquitination phase through addition of the Rpn11 inhibitor 1,10-phenanthroline (*o*PA; Fig. 3D) (*27*). To distinguish between potential effects of ubiquitin binding and substrate degradation on proteasome conformations, we used a previously developed ubiquitin-independent substrate delivery system. In this system, the bacterial adaptor SspB is fused to the N terminus of the Rpt2 ATPase subunit, which allows the recruitment and degradation of nonubiquitinated substrates that contain the SspB-interacting ssrA tag in their unstructured initiation region (fig. S10A) (*52*). Apparent FRET efficiency traces in the conformational change assay revealed that proteasomes with SspB-delivered or ubiquitinated substrates exhibit similar prolonged dwells in the high-FRET state that likely represent individual substrate-processing events (Fig. 4A and fig. S10, B to D). This interpretation is supported by the observed correlation between the length of these high–FRET efficiency dwells and the thermodynamic stability of the substrate’s I27 domain. SspB-delivered wild-type I27 substrate shows significantly longer processing dwells (τ_deg_ = 47 ± 4 s) than the I27^V15P^ single-mutant (τ_deg_ = 10.7 ± 0.6 s) or the least stable I27^V13P/V15P^ double-mutant substrate (τ_deg_ = 7.3 ± 0.2 s; Fig. 4, A and B, fig. S11, and table S4). Given that the conformational switch to high-FRET non-s1 states is induced by substrate engagement, it can be assumed that the proteasome stably switches back to the s1 state after translocation has been completed and the substrate terminus has cleared the ATPase ring on its way into the 20*S* core peptidase. The high–FRET efficiency dwells in the conformational change assay therefore represent an accurate readout for the total time of substrate processing after engagement, and the derived time constants for all three substrate variants are in excellent agreement with previous fluorescence anisotropy–based measurements of single-turnover degradation in bulk (*4*). Although our data did not allow the direct calculation of unfolding time constants, we could estimate them based on the times required for complete processing and for translocation, assuming that these degradation steps are irreversible because of their coupling to multiple ATP hydrolysis events. On the basis of a velocity of ~30 amino acids s^−1^ for unobstructed translocation, it would take ~4.6 s to completely thread the ~138 residues that reside above or within the ATPase ring after engagement of our model substrates (fig. S1B). The unfolding times could thus be estimated as τ_unfold_ ~42 s for wild-type I27, τ_unfold_ ~6 s for I27^V15P^, and τ_unfold_ ~3 s for I27^V13P/V15P^.

The high–FRET efficiency dwells during substrate processing show intermittent, brief returns to the low-FRET s1 state, with a frequency that depends on the thermodynamic stability of the substrate (Fig. 4A and fig. S11). While the strongly destabilized I27^V13P/V15P^ variant shows barely any of these transitions to the s1 state at our experimental time resolution (50 ms), they are more frequent (0.7 ± 0.2 per processing) for I27^V15P^ and very prominent (11 ± 2 per processing) during the motor’s attempts to unfold wild-type I27 (table S5). However, for all substrate variants, the s1 returns are noticeably absent from the last 3 to 5 s of processing (Fig. 4A and fig. S11, B and C), which likely reflects the unobstructed translocation of the substrate polypeptide after successful unfolding and is consistent with the translocation rate that we determined above. Intermittent s1 returns during the unfolding phase may thus represent slippage events, where the pore loops struggle with a high unfolding energy barrier and lose grip of the polypeptide, leading to a transient elimination of the substrate-induced stabilization of non-s1 states. This slippage would be in agreement with our previous findings that ATP hydrolysis, and presumably motor movements, does not cease when substrate translocation stalls at an unfolding barrier (*12*). The uninterrupted high–FRET efficiency dwells during processing of I27^V13P/V15P^ or translocation of the unfolded polypeptides contradict a ratcheting mechanism, in which substrate translocation is driven by repetitive transitions between s1 and non-s1 states (*29*). Instead, the proteasome appears to switch out of the s1 state upon substrate engagement and then cycle through the various non-s1 states with different spiral staircase arrangements of ATPase subunits to propel substrates by some kind of hand-over-hand mechanism, not requiring any returns to s1 (*35*, *36*). We also observed that ΔRpn10 and Rpn5^VTENKIF^ mutant proteasomes exhibit high–FRET efficiency dwells during I27^V13P/V15P^ substrate degradation that are of similar persistence and length (τ_deg_ = 7.9 ± 0.3 and 8.4 ± 0.3 s, respectively) as those of wild-type proteasomes (fig. S10, B and C). Although these mutations shift the proteasome conformational equilibrium in the absence of substrate, they do not affect processing once a substrate is engaged, which is in agreement with our model that Rpt pore loop interactions with the substrate polypeptide dominate the conformational equilibrium over lid-base contacts or the effects of ATP binding and hydrolysis by the Rpt motor.

### Ubiquitin slows conformational switching and accelerates degradation

**Fig. 5.**
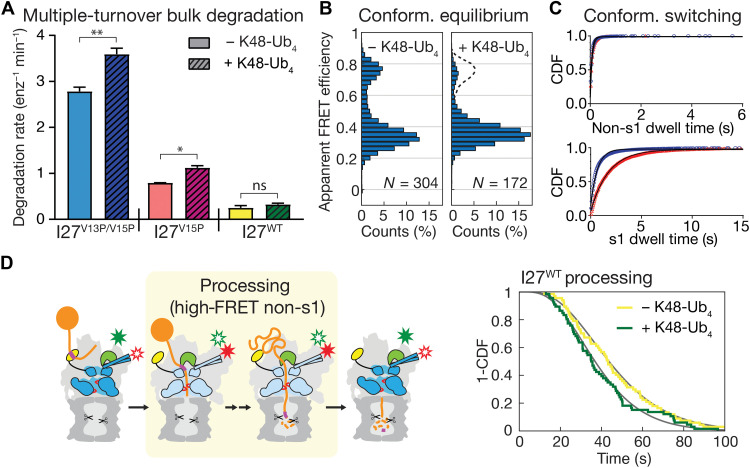
Ubiquitin chains modulate conformational dynamics and activate the proteasome. (**A**) Rates for the multiple-turnover degradation of the I27^V13P/V15P^, I27^V15P^, and I27^WT^ substrates after SspB-mediated delivery to the proteasome in the absence (solid bars) and presence (hatched bars) of 10 μM unanchored K48-Ub_4_ (*N* = 3 technical replicates). Statistical significance was calculated using an unpaired two-tailed Welch’s *t* test: **P = 0.0091; *P = 0.0185; ns, *P* = 0.2766. (**B**) Histograms for the FRET state distributions of wild-type proteasomes in the absence (left) and presence (right) of K48-Ub_4_. The dashed line on the right shows the distribution in the absence of K48-Ub_4_ for comparison. (**C**) Dwell time distributions for the high-FRET non-s1 states (top) and low-FRET s1 state (bottom) during the conformational switching of the substrate-free proteasome in the absence (blue, *N* = 197) and presence of K48-Ub_4_ (red, *N* = 119), with fits to single exponentials shown as black lines. (**D**) Times for the substrate-processing dwells during degradation of SspB-delivered I27^WT^ (yellow highlight in the schematic, left) are shown as survival plots (right) for the absence (yellow, *N* = 67) and presence of K48-Ub_4_ (green, *N* = 66). Survival plots were compared using the Mantel-Cox test, yielding a *P* value of 0.0844.

**Fig. 6.**
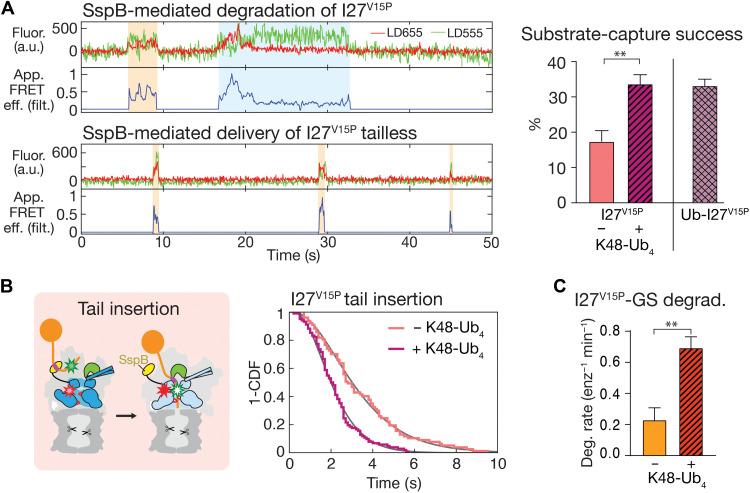
Ubiquitin effects on substrate capturing and engagement. (**A**) Top left: Example traces for the substrate-processing assay monitoring SspB-mediated degradation of nonubiquitinated I27^V15P^ substrate after excitation at 532 nm, with the raw donor fluorescence in green, raw acceptor fluorescence in red, and the corresponding filtered apparent FRET efficiency in blue. The traces show two substrate-binding events, with the first one (orange shading, intermediate apparent FRET efficiency) being nonproductive and the second (blue shading) leading to degradation. Bottom left: Example traces for a tailless nonubiquitinated I27^V15P^ substrate that cannot engage with the ATPase motor and exhibits only nonproductive binding events with intermediate apparent FRET efficiency (orange shading). Right: Substrate-capture success of the proteasome, as determined by the fractional rate of productive substrate binding and degradation events relative to all substrate encounters, for SspB-delivered nonubiquitinated I27^V15P^ in the absence (red) and presence (hatched magenta) of unanchored K48-Ub_4_ (*N* = 5 technical replicates, with each contributing 296 to 470 substrate-capture events), and in comparison to ubiquitinated I27^V15P^ (Ub-I27^V15P^crosshatched purple). Statistical significance was calculated using the Mann-Whitney test, with **P = 0.0079. (**B**) Left: Schematic for the tail insertion of SspB-delivered substrate. Right: Survival plots for the tail-insertion times of SspB-delivered I27^V15P^ substrate in the absence (red, *N* = 77) and presence (magenta, *N* = 102) of K48-Ub_4_. Fit lines in gray are based on the cumulative distribution of the gamma function derived from fitting the dwell time distributions. Comparing the survival plots using the Mantel-Cox test revealed *P* < 0.0001. (**C**) Rates for the multiple-turnover degradation of the I27^V15P^-GS substrate after SspB-mediated delivery to the proteasome in the absence (solid bar) and presence (hatched bar) of 10 μM K48-Ub_4_ (*N* = 3 technical replicates). Statistical significance was calculated using an unpaired two-tailed Welch’s *t* test: **P = 0.0017.

A major advantage of our SspB-mediated substrate delivery system is that it allows us to directly measure potential allosteric effects of ubiquitin chain binding to the proteasome on substrate degradation and the conformational dynamics. In bulk measurements, we observed a 1.3- to 1.4-fold acceleration of titin substrate degradation by unanchored K48-linked ubiquitin tetramers (K48-Ub_4_; Fig. 5A and figs. S12 and S13, A to C), which is consistent with a previous study reporting that substrate-attached ubiquitin chains affect proteasomal degradation (*42*). To determine which steps of degradation were modulated by ubiquitin chain binding to the proteasome, we first analyzed the effects of unanchored K48-Ub_4_ in our FRET-based conformational dynamics assay. In the absence of substrate, addition of K48-Ub_4_ biases the proteasome conformational equilibrium toward the s1 state (Fig. 5B and figs. S14 and S15A). Linear and K63-linked ubiquitin chains showed similar effects (table S3), suggesting that diverse ubiquitin linkage types induce this common conformational response. Ubiquitin chains increase the dwell time of the s1 state and thus reduce the rate of the s1–to–non-s1 transition by ~3-fold, from 1.2 to 0.4 s^−1^, while having minimal effects on the return from non-s1 to the s1 state (Fig. 5C and table S3). To assess whether ubiquitin chains modulate substrate unfolding and translocation, we used our FRET-based conformational change assay with SspB-fused proteasomes. In the presence of K48-Ub_4_ chains, we observed high–FRET efficiency dwells of τ_deg_ = 40 ± 4 s for wild-type I27, τ_deg_ = 9.0 ± 0.3 s for I27^V15P^, and τ_deg_ = 7.0 ± 0.1 s for I27^V13P/V15P^, which are 15 to 5% shorter than in the absence of ubiquitin (Fig. 5D and table S4). Although there appears to be a slight trend toward faster processing in the presence of ubiquitin that correlates with the thermodynamic stability of the substrate folded domain, those effects are very minor and statistically insignificant. The presence of ubiquitin chains has also no discernable effects on the proteasome conformational dynamics during substrate processing (fig. S16 and table S5) and may thus facilitate degradation primarily through faster engagement, rather than accelerated unfolding and translocation (fig. S15C). Ubiquitin binding stabilizes the proteasome in the engagement-competent s1 conformation with a well-accessible central channel (Fig. 5C), and we therefore hypothesized that it promotes substrate insertion and degradation initiation. To test this, we used the FRET-based substrate-processing assay for monitoring SspB-mediated I27^V15P^ degradation and analyzed the engagement efficiency or “capture success rate,” which is reflected by the number of complete processing events per total number of substrate encounters (Fig. 6A). The presence of ubiquitin chains nearly doubles this capture success, from 17 ± 3 to 34 ± 3% (Fig. 6A), a value that is very similar to the capture success for the ubiquitinated substrate (33 ± 2%). Furthermore, we observed that proteasome-bound ubiquitin chains accelerate substrate tail insertion, lowering the time constant from 3.4 to 2.2 s, which approaches the value for ubiquitinated substrates (Fig. 6B, fig. S17, and table S1). This confirmed our hypothesis that the ubiquitin-mediated increase in lifetime of the s1 state to 2.5 s (*k*_s1_ = 0.4 s^−1^) and, thus, into the range of substrate tail insertion kinetics facilitates degradation initiation and increases capture success. The SspB-fused proteasomes used for these substrate-processing assays show a s1–to–non-s1 transition rate in the absence of ubiquitin chains that is ~30% lower than for wild-type proteasomes (*k*_s1_ = 0.8 s^−1^ versus 1.2 s^−1^; fig. S14 and table S3), possibly due to steric effects, yet an identical rate of 0.4 s^−1^ in the presence of ubiquitin. The s1 state lifetime of SspB-fused proteasomes is therefore only twofold extended by ubiquitin chain binding, whereas wild-type proteasomes show a threefold effect, which is expected to cause an even stronger ubiquitin-mediated acceleration of substrate tail insertion than observed here with the SspB fusion. Substrate capture by the proteasome depends on the competition between the engagement of the polypeptide by the ATPase motor and the dissociation of a substrate-attached ubiquitin chain from proteasomal receptors or, for our ubiquitin-independent delivery system, the dissociation of the ssrA peptide motif from the proteasome-fused SspB adaptor. We measured the time constant for the dissociation of a tailless, ubiquitinated substrate from proteasomal receptors as τ_Ub-off_ = 0.61 ± 0.12 s (fig. S15B), and a time constant of τ_ssrA-off_ = 0.32 s was previously determined for the dissociation of ssrA from SspB (*53*). Both dissociations occur much faster than the complete tail insertion and motor engagement of a ubiquitinated substrate (τ_ins_ = 1.8 s) or an SspB-delivered substrate in the presence of unanchored ubiquitin chains (τ_ins_ = 2.2 s). We therefore propose that a substrate’s flexible initiation region rapidly forms stabilizing interactions with the upper part of the processing channel that can prevent immediate substrate dissociation from the proteasome. This model also agrees with our previous findings that the presence of a flexible initiation region increases the apparent substrate affinity for the proteasome by ~10-fold (*4*). The initial interactions with the channel are likely not stable enough to withstand a collision of the substrate polypeptide with Rpn11 when the proteasome transitions from the s1 to non-s1 states, in which Rpn11 obstructs the central pore and leaves only a small gap above the N-ring for substrates to be translocated through (Figs. 1A and 3A). Successful substrate capture probably depends on a stable engagement with the pore loops of the ATPase motor before the proteasome switches to non-s1 states, which would explain why a ubiquitin-induced longer time constant for conformational switching facilitates substrate tail insertion and engagement, and causes an increase in the capture success rate. This faster engagement is likely responsible for the moderate 1.3- to 1.4-fold acceleration of degradation for our titin model substrates that bear an ideal, cyclin B–derived initiation region of 35 residues (Fig. 5A). However, most of physiological substrates are expected to contain shorter or less ideal initiation regions and may therefore be harder to engage or enter the proteasome more slowly. To better mimic these endogenous substrates and further explore the effects of ubiquitin on degradation initiation, we generated a titin^V15P^ variant in which the C-terminal 10 amino acids of the 35-residue tail were replaced with a glycine-serine–rich sequence whose low complexity was previously shown to slow initiation (fig. S1B) (*4*, *54*). The presence of K48-Ub_4_ chains causes a stronger, threefold acceleration of degradation for this more challenging titin^V15P^-GS substrate (Fig. 6c), consistent with our findings that ubiquitin stabilizes the engagement-competent proteasome state, gives the substrate more time for insertion into the ATPase motor, and thus increases substrate capture success.

## DISCUSSION

Our single-molecule FRET-based assays revealed that the proteasome’s dynamic switching between the engagement-competent s1 state and the processing-competent non-s1 states controls access to the central channel and represents a selectivity filter for appropriate substrates that can rapidly enter and engage the ATPase motor. Ubiquitin chain binding to the proteasome functions as an allosteric regulator that slows down this conformational switching, stabilizes the s1 state, facilitates insertion of the substrate’s flexible initiation region, and consequently increases the success rate of capturing a substrate for degradation.

Upon substrate engagement, the proteasome switches to the non-s1 states for substrate unfolding and translocation, which occurs at a velocity of ~30 amino acids s^−1^ and thus about an order of magnitude faster than previously assumed based on bulk ATPase measurements and the translocation step size derived from substrate-bound structures. Cotranslocational ubiquitin chain removal by Rpn11 also happens very rapidly, at a rate ~50-fold higher than the maximal rate for translocation-independent ubiquitin cleavage by Rpn11 (*27*). Deubiquitination therefore does not contribute to the overall degradation time, even for substrates with multiple ubiquitin modifications (*4*). The time for mechanical unfolding correlates with the substrate’s thermodynamic stability and is only insignificantly affected by the presence of ubiquitin chains, at least for our model substrates. Contrary to a recently proposed model (*55*), the proteasome’s conformational dynamics or the relative stabilities of the engagement-competent s1 versus processing-competent non-s1 states do not seem to play a role in determining the kinetics of unfolding and translocation, and ubiquitin chains accelerate degradation primarily by helping substrate engagement. Unanchored ubiquitin chains thereby cause a moderate increase in the degradation rates for substrates with ideal initiation regions, but lead to a considerable threefold acceleration for a harder-to-engage substrate variant with a slippery tail. Ubiquitin’s allosteric effects on degradation are expected to be even larger for covalently attached chains and provide a significant advantage especially in the cellular environment, where ubiquitinated substrates have to compete with nonubiquitinated proteins that may be present at high local concentrations near the proteasome due to molecular crowding. In summary, our single-molecule FRET measurements reveal important new details about ubiquitin-dependent substrate processing by the proteasome and lay the foundation for further studies of how AAA^+^ motors engage, translocate, and unfold their substrates and how effectors such as ubiquitin chains allosterically regulate these processes.

## MATERIALS AND METHODS

### Purification and labeling of the lid and base subcomplexes

Recombinant proteasomes were purified, fluorescently labeled, and reconstituted as described previously (*4*, *56*). Three separate purifications (base, lid, and core) are required to reconstitute 26*S* recombinant proteasomes. For recombinant expression of the yeast base subcomplex, *Escherichia coli* BL21-Star (DE3) (Invitrogen) was cotransformed with the plasmids pAM81, pAM82, and pAM83 (derived from pETDuet, pCOLADuet, and pACYCDuet, respectively), which code for Rpt1 to Rpt6 (including an amber codon TAG at the desired position for site-specific unnatural amino acid incorporation); Rpn1, Rpn2, and Rpn13; the four base assembly chaperones Rpn14, Hsm3, Nas2, and Nas6; and rare tRNAs. An additional fourth plasmid, pUltra_pAzFRS.2.t1_UAG-tRNA, coded the AzF tRNA synthase/tRNA to allow unnatural amino acid incorporation. Cells were grown at 37°C in 6 liters of terrific broth (Novagen) to an optical density at 600 nm (OD_600_) between 0.6 and 0.9, then pelleted and resuspended in 1 liter of terrific broth containing 2 mM AzF (Amatek Chemical), incubated at 30°C for 30 min, induced with 1 mM isopropyl-β-d-thiogalactopyranoside (IPTG) for 5 hours at 30°C, and then further incubated overnight at 16°C. Cells were pelleted and resuspended in lysis buffer [60 mM Hepes (pH 7.6), 100 mM NaCl, 100 mM KCl, 10 mM MgCl_2_, 5% glycerol, and 20 mM imidazole] supplemented with 1 mM ATP, lysozyme, benzonase (Novagen), and protease inhibitors (aprotinin, leupeptin, pepstatin, and phenylmethylsulfonyl fluoride). The cells were lysed by sonication, the lysate was clarified by centrifugation for 30 min at 30,000*g*, and the base subcomplex was fluorescently labeled and purified by a three-step procedure, with 0.5 mM ATP present in all buffers. First, the His-Rpt3 containing complexes were isolated using a 5-ml HisTrap FF crude column (Cytiva), and then fully assembled base complexes containing Flag-Rpt1 were selected for using anti-Flag M2 affinity resin (Sigma-Aldrich). After concentration (Amicon Ultracel-100K), the base subcomplexes were incubated with 150 μM 5,5′-dithiobis-(2-nitrobenzoic acid) (DTNB) for 10 min at room temperature to reversibly block surface-exposed cysteines before the AzF (incorporated in place of Q49 of Rpt5 or I191 of Rpt1) was reacted with 300 μM dibenzocyclooctyne (DBCO)–conjugated LD655 dye (a sulfo-Cy5 derivative from Lumidyne Technologies; the DBCO modification is custom synthesis) at 4°C overnight. The reaction was quenched with 1 mM free AzF, followed by addition of 5 mM dithiothreitol (DTT) to reverse the DTNB modification of cysteines. Last, labeled base subcomplexes were purified using size exclusion chromatography with a Superose 6 Increase 10/300 column (Cytiva) equilibrated in GF buffer [30 mM Hepes (pH 7.6), 50 mM NaCl, 50 mM KCl, 10 mM MgCl_2_, 5% glycerol, 0.5 mM ATP, and 0.5 mM tris(2-carboxyethyl)phosphine (TCEP)]. Base concentrations were determined by Bradford assay, and labeling efficiencies were determined to be ~70 to 90% according to absorbance of the fluorophore.

Recombinant yeast lid was expressed from three plasmids, pAM80, pAM85, and pAM86, that code for Rpn3, Rpn5 to Rpn9, Rpn11, Rpn12, Sem1, and rare tRNAs; purified using a three-step procedure; and fluorescently labeled, as previously described (*4*, *56*). Briefly, fully assembled complexes containing His-Rpn12 and maltose-binding protein fused Rpn6 (MBP-Rpn6) were purified using a HisTrap and amylose resin [New England Biolabs (NEB)], and the MBP tag was cleaved with Human Rhinovirus (HRV) protease. Subsequent labeling of the AzF (incorporated in place of F2 of Rpn9) with LD555-DBCO (a sulfo-Cy3 derivative from Lumidyne Technologies; the DBCO modification is custom synthesis) and the purification by size exclusion chromatography proceeded in a similar fashion as for the base subcomplex. Lid labeling efficiencies were ~60 to 80%.

The ubiquitin receptor subunit Rpn10 was expressed and purified separately as previously described (*4*). Pelleted cells were resuspended in lysis buffer supplemented with 20 mM imidazole, lysozyme, benzonase, and protease inhibitors and lysed by sonication. After clarification, the lysate was batch bound to Ni–nitrilotriacetic acid (NTA) affinity resin (Thermo Fisher Scientific), the resin was washed with lysis buffer, and proteins were eluted with lysis buffer containing 250 mM imidazole. Rpn10 was further purified by size exclusion chromatography using a Superdex 75 16/60 column (Cytiva). The ubiquitin receptor subunit Rpn13 was expressed and purified similar to Rpn10, with the added step of incubation with HRV protease at 25°C for 1 hour to remove a cleavable histidine tag before size exclusion chromatography.

### Cloning and purification of 20*S* core particle

A sequence encoding for an AviTag followed by a 3xFLAG tag was cloned into the C terminus of the endogenous Pre1 gene of a W303 yeast strain. The purification of AviTag-20*S* core was performed similar to a procedure previously described (*56*). Briefly, the yeast strain was grown in yeast extract, peptone, and dextrose at 30°C for 3 days. The cells were pelleted, resuspended in lysis buffer [60 mM Hepes (pH 7.6), 500 mM NaCl, 1 mM EDTA, and 0.2% NP-40], popcorned into liquid nitrogen, and then lysed in a Cryomill 6875D (SPEX SamplePrep). The lysate was allowed to return to room temperature and clarified by centrifugation, and the 20*S* core particle was purified using anti-Flag M2 affinity resin (Sigma-Aldrich). The elution was concentrated using an Amicon Ultracel-100K, and the concentration was determined by absorbance at 280 nm. After biotinylation through incubation with 25 μM E. coli biotin ligase BirA and 100 μM biotin in the presence of 10 mM ATP and 10 mM MgCl_2_ overnight at 4°C, the core was further purified by size exclusion chromatography with a Superose 6 Increase 10/300 column (Cytiva), equilibrated in GF buffer [30 mM Hepes (pH 7.6), 50 mM NaCl, 50 mM KCl, 10 mM MgCl_2_, 5% glycerol, and 0.5 mM TCEP]. The extent of biotinylation was determined in a gel shift assay by incubation with NeutrAvidin (Thermo Fisher Scientific).

### Substrate purification and labeling

Titin I27 substrate expression was performed as described previously (*4*), using a plasmid that codes for the desired protein sequence fused to an C-terminal intein and chitin binding domain as part of the IMPACT purification system (NEB). Two liters of cells were grown to an OD_600_ of 0.6 and induced with 0.5 mM IPTG for 3 hours at 30°C. Cells were pelleted, resuspended in chitin binding buffer [60 mM Hepes (pH 7.6), 150 mM NaCl, 1 mM EDTA, and 5% glycerol] with benzonase and protease inhibitors, and lysed. After lysate clarification by centrifugation, the protein was batch bound to 10 ml of chitin resin (NEB) for 1 hour at 4°C. The resin was washed with 100 ml of chitin binding buffer, resuspended in cleavage buffer [30 mM Hepes (pH 8.5), 100 mM NaCl, 1 mM EDTA, 5% glycerol, and 50 mM DTT], and incubated overnight at 4°C. The flowthrough containing the cleaved protein was collected, run over fresh chitin resin to remove any uncleaved protein, concentrated, and further purified using a Superdex 75 16/60 column (Cytiva) equilibrated with GF buffer. Fluorophores were covalently attached either to the substrates’ N terminus for bulk degradation measurements or to single engineered cysteines for FRET-based single-molecule experiments. For N-terminal labeling with fluorescein, we used sortase A (*4*, *57*) and the peptide FAM-HHHHHHLPETGG (GenScript) and enriched the labeled substrates using Ni-NTA agarose beads (Thermo Fisher Scientific) to ensure 100% labeling efficiency. For the labeling of engineered cysteines with sulfo-Cy3 maleimide (Lumiprobe Corporation, catalog no. 21380) or LD555 maleimide (Lumidyne Technologies, catalog no. 04), substrates were dialyzed into labeling buffer [30 mM Hepes (pH 7.2), 150 mM NaCl, and 1 mM EDTA] for 3 hours at room temperature using Slide-A-Lyzer mini dialysis cups (Thermo Fisher Scientific), diluted to 100 μM, and then incubated for 1 hour at room temperature with 200 μM fluorophore. Excess dye was quenched with 1 mM DTT and removed by size exclusion chromatography on a Superdex 75 10/300 column equilibrated with GF buffer. The cysteine-based labeling efficiency was ~50 to 60%. Initial batches of the titin I27^V13P/V15P^ substrate were labeled with the sulfo-Cy3 dye, while later batches and all other substrates were labeled with LD555, which made no difference for our measurements other than extending the lifetime of the donor dye.

### Ubiquitin purification, substrate ubiquitination, and preparation of unanchored ubiquitin chains

*E. Coli* Bl21*(DE3) cells were transformed with a pET28a plasmid coding for yeast ubiquitin, grown in terrific broth at 37°C until OD_600_ = 0.6 to 0.9; induced with 1 mM IPTG overnight at 18°C; pelleted; resuspended in lysis buffer with lysozyme, benzonase, and protease inhibitors; and lysed by sonication. The lysate was clarified by centrifugation, and its pH was adjusted to 4.5 using acetic acid. Protein precipitate was removed by centrifugation, and the supernatant was dialyzed against 50 mM Na-acetate (pH 4.5) overnight at 4°C. Ubiquitin was purified by cation-exchange chromatography with a 5-ml HiTrap SP FF column (Cytiva) and a gradient of 0 to 0.5 M NaCl in 50 mM Na-acetate (pH 4.5), followed by size exclusion chromatography using a Superdex 75 16/60 column equilibrated with GF buffer. Enzymatic addition of ubiquitin chains to substrates containing PPPYX motifs and a single lysine residue was performed as previously described (*4*), incubating 10 μM substrate for 3 hours at 25°C in GF buffer with 10 mM ATP, 400 μM ubiquitin, 2.5 μM mouse E1, 2.5 μM Ubc1, and 25 μM Rsp5. K48-Ub_4_ and K63-Ub_4_ were synthesized and purified as previously described (*58*). K48-linked chains were synthesized by incubating 1 mM ubiquitin with 1 μM mE1, 5 μM Cdc34, and 10 mM ATP in GF buffer overnight at 37°C. K63-linked chains were synthesized by incubating 1 mM ubiquitin with 1 μM mE1, 2.5 μM Ube1a/UbcH13, and 10 mM ATP in GF buffer overnight at 37°C. Tetrameric ubiquitin chains were separated from the other chain lengths using a Resource S cation-exchange column (Cytiva) with a 0 to 600 mM NaCl gradient and a Superdex 75 increase 10/300 (Cytiva) in GF buffer and were verified by gel electrophoresis. Linear tetra-ubiquitin chains were prepared as previously described (*4*): A linear hexa-His SUMO (small ubiquitin-like modifier)–tetra-ubiquitin fusion was affinity purified using Ni-NTA, cleaved with the protease SENP2, and then purified over a Superdex 75 16/60 (Cytiva) in GF buffer.

### Microscope setup

Single-molecule data acquisition was performed at room temperature on a custom-built objective-type TIRF microscope (fig. S2A), similar to a design described previously (*59*). The microscope is built on top of a Nikon Eclipse Ti turret that is equipped with an ultrastable single-molecule stage (model KS-N) and a 60× Apo TIRF (1.49 numerical aperture) oil objective (Nikon), which we used with Cargille DF immersion oil. No active feedback stabilization was used, as the stage exhibited a drift of less than 0.5 pixels over 20 min (more than 10 times longer than a normal acquisition). Donor dyes (Cy3 and LD555) were imaged using a Coherent OBIS LS 100-mW 532-nm solid-state laser (operated at 2- to 10-mW power). Acceptor dyes (LD655) were imaged using a Uniphase 10-mW 633-nm helium-neon laser (operated at 10 mW and attenuated to ~5 mW using a neutral density (ND) filter). All lasers were steered through Galilean beam expanding optics to save space. The collimated beams were focused using an achromatic doublet (*f* = 100 mm) onto a conjugate back focal plane of the objective that was relayed outside of the microscope with a Ti-Lapp branch (Nikon). The focusing lens was coupled to a positioning stage and mirror assembly, such that the TIRF angle could be adjusted by moving the lens and mirror simultaneously. We used a filter cube from Chroma (TRF59907-EM). The emission from donor and acceptor dyes were separated using image splitting optics (W-View Gemini from Hamamatsu) equipped with a T640LPXR-UF2 dichroic mirror and ET595/50-m and ET690/50-m bandpass filters (Chroma) and simultaneously imaged with the same camera (Andor iXon 512 × 512 EM charge-coupled device DU-897). For registration of the two emission channels, images were acquired with 100-nm TetraSpeck beads (Thermo Fisher Scientific). For preparation of the TetraSpeck bead sample, poly-d-lysine (2 mg/ml) was flowed into a reaction chamber and incubated for 3 min, followed by a wash with GF buffer before flowing in a 1:50 dilution of TetraSpeck beads and incubation for 5 min. Excess beads were washed out using GF buffer, and the sample was imaged using the 532-nm laser. Registration was then performed by overlaying cropped regions from the donor and acceptor emission channels such that the bead positions in each channel agreed. All acquisitions were performed with the following camera settings: EM gain at 60, readout rate at 17 MHz, and preamp set to 3, with activated overlap mode and camera internally triggered.

### Single-molecule fluorescence microscopy data acquisition

Reaction chambers were assembled on microscope slides using double-sided Scotch tape and polyethylene glycol (PEG)–coated coverslips with low-density PEG-biotin (MicroSurfaces Inc.). The reaction chambers were incubated with NeutrAvidin (0.05 mg/ml; Thermo Fisher Scientific), and excess NeutrAvidin was removed by washing with 50 μl of assay buffer [GF buffer supplemented with 0.3 μM Rpn10, 1.2 mM Trolox, 0.2 mM β-mercaptoethanol, 2 mM ATP, and an ATP regeneration system consisting of creatine kinase (0.03 mg/ml) and 16 mM creatine phosphate]. The assay buffer for the conformational change assay during substrate processing was further supplemented with 0.3 μM Rpn13 to assure complete proteasome assemblies and because this subunit was previously reported to be required for unfoldase activation (*60*). Proteasomes were reconstituted by incubating 600 nM lid, 400 nM base, and 500 nM biotinylated core with 1 μM Rpn10 in GF containing 2 mM ATP and the ATP regeneration system for 10 min at room temperature, similar to previously described reconstitutions (*4*). The reconstituted, dye- and biotin-labeled proteasomes were tested in bulk assays for full degradation activity (*4*), diluted to ~100 pM, flowed into the reaction chamber, and allowed to incubate for 5 min. The extent of surface deposition was controlled by adjusting the proteasome dilution such that single fluorescent molecules could clearly be distinguished. Excess proteasomes in solution were washed out with 50 μl of assay buffer, followed by 50 μl of imaging buffer [assay buffer supplemented with the protocatechuic acid (PCA)/protocatechuate-3,4-dioxygenase (PCD) oxygen scavenging system described previously (*61*)]. The use of PEG-coated coverslips ensured minimal nonspecific binding of proteasome subcomplexes or subunits in the absence of biotinylated core particle (fig. S2C). Substrate was added to the imaging buffer for assays that required it. For the conformational change assay, saturating the amount of unlabeled substrate was added at a concentration of either 400 nM for ubiquitinated substrate or 5 μM for SspB-delivered substrate. For the substrate-processing assay, Cy3- or LD555 donor–labeled substrate was added at low concentrations of 25 to 80 nM to minimize interfering background fluorescence of unbound substrate molecules in the TIRF experiments. To approximate the number of amino acids translocated during the FRET-decay phase of the substrate-processing assays, we generated substrate variants with the donor dye attached in various positions of the C-terminal flexible tail and stalled their translocation in a defined position using proteasomes with the catalytically dead Rpn11^AXA^ mutant (*35*). After a 5-min incubation of the substrates and proteasomes in the presence of 2 mM ATP and an ATP regeneration system on the coverslip, excess substrates in solution were washed away with 50 μl of assay buffer, an imaging buffer containing 2 mM ATPγS was flowed in to further reduce the minor movement of stalled substrates due to the ATPase motor activity, and particles were imaged after a 5-min incubation. For all single-molecule assays, acceptor fluorophores were imaged first by taking a single-frame snapshot following a 633-nm laser excitation. Both donor and acceptor fluorescence signals were simultaneously monitored following a 532-nm laser excitation.

### Data analysis

For both the conformational dynamics and substrate-processing assays, we analyzed only singly capped and singly labeled proteasomes, which were identified on the basis of their total fluorescence and a single photobleaching step. Raw images from substrate-processing acquisitions were cropped, spatially aligned using registration coordinates derived from analyzing TetraSpeck images, and parsed with the ImageJ plugin spotIntensityInAllChannels (*62*). The parsed data were then sorted and analyzed using a custom-built MATLAB app, which can be found on GitHub and Zenodo (https://github.com/jabard89/tirfexplorer and https://doi.org/10.5281/zenodo.7255657). First, the raw donor and acceptor fluorescence traces were smoothed with a moving average filter—MATLAB’s smooth function with a heuristically determined averaging window of five frames (equivalent to 250 ms), and the acceptor fluorescence was further corrected for donor bleedthrough. “Filtered apparent FRET efficiency” was calculated as *I*_A_/(*I*_D_ + *I*_A_), where *I*_D_ is the filtered donor fluorescence signal and *I*_A_ is the filtered and bleedthrough-corrected acceptor fluorescence signal (fig. S2D). The start and end of each substrate-processing event were determined from the total fluorescence, which depends on the residence time of a donor-labeled substrate, and the apparent FRET efficiency values outside substrate processing events were not calculated (indicated by dashed lines in all related figures) for easier scoring of substrate processing events.

Parsed, processed, and filtered substrate processing traces were inspected manually and scored for various features. The tail insertion phase was measured from the appearance of a donor signal and intermediate apparent FRET efficiency to the point of the first high–FRET efficiency peak. The decay of apparent FRET efficiency was scored similarly but in reverse, i.e., by measuring the time from a high–FRET efficiency peak (~0.82) to the point where the value reached ~0.32. The length of the high–FRET efficiency phase was determined as the time between the tail insertion (FRET-increase) phase and the translocation (FRET-decay) phase. Integration time for these measurements was 100 ms, which also sets a limit for the processes that we can distinguish. The distributions of each measurement (tail insertion, high-FRET phase, etc.) were fit to a gamma distribution using MATLAB’s fitdist function. The parameters of the fit were then used to generate fit lines for one–cumulative distribution functions (1-CDF) using MATLAB’s gamcdf function. Empirically derived 1-CDF plots were generated from the measurements using MATLAB’s ecdf function. To determine the substrate-capture success of the proteasome in substrate-processing assays, we calculated the fractional number of productive binding events leading to degradation relative to the total number of substrate encounters (both productive and nonproductive). We considered a substrate binding event when an increase in donor fluorescence colocalized with acceptor fluorescence. Binding events were scored as productive and indicative of degradation if they exhibited a high-FRET phase followed by a donor fluorescence recovery and extended donor fluorescence dwell as a measure for successful threading. Binding events were scored as nonproductive if the intermediate FRET efficiency was not followed by an increase to high–FRET efficiency or a recovery of the donor fluorescence. A tailless titin I25^V15P^ substrate that cannot get engaged by the ATPase motor (*4*) was used to show that the short intermediate FRET efficiency events represented nonproductive binding (Fig. 4E). Five movies, containing at least 250 binding events per movie, were analyzed for each condition, and the error is the SD for the five measurements.

The conformational dynamics assays were performed at a sampling rate of 20 Hz, except during the degradation of I27^WT^, for which the sample rate was reduced to 5 Hz to capture the entire substrate-processing events before photobleaching. Raw images were cropped and spatially aligned, and movies were parsed by the Spartan software package (*63*). Once parsed, the traces were sorted, plotted with Spartan’s built-in functions, and analyzed using hidden Markov modeling with ebFRET (*64*). The dwell time distributions for each FRET state were extracted and fit to a single exponential, with errors representing 95% confidence interval of the fit. Substrate-processing dwells were scored manually from the onset to the stable end of high apparent FRET efficiency, including brief excursions to low–FRET efficiency during the processing of the I27^V15P^ and I27^WT^ substrates.

### Fluorescence polarization-based multiple-turnover degradation assay

Proteasomes were reconstituted at 2× concentration by mixing and incubating 100 nM core particle, 800 nM SspB-fused base, 1.2 μM lid, and 2 μM Rpn10 at room temperature for 10 min in the presence of an ATP regeneration system [creatine kinase (0.03 mg/ml) and 16 mM creatine phosphate]. Substrate mix was prepared at 2× concentration (20 μM) in GF buffer supplemented with bovine serum albumin (1 mg/ml) and 10 mM ATP. For the experiments with K48-Ub_4_, 20 μM chains were added in the substrate mix. The multiple-turnover degradation reaction was initiated by mixing 7 μl of 2× reconstituted SspB-fused proteasome and 7 μl of 2× substrate mix. Ten microliters of the reaction was immediately transferred to a 384-well low-volume black flat bottom plate (Corning, #3820) and prewarmed to 30°C, and degradation was monitored by the loss of fluorescence polarization signal from 5-carboxyfluorescein–labeled substrates in a CLARIOstar Plus plate reader (BMG Labtech) at 30°C. Rates for multiple-turnover degradation were determined by linear regression of the initial change in polarization and normalizing the fit with the measured differences in fluorescence polarization signals of undegraded substrates and substrates fully degraded by chymotrypsin (0.1 μg/μl).

## Notes

View/request a protocol for this paper from *Bio-protocol*.
