# Supplementary material for: Ubiquitin modulates 26S proteasome conformational dynamics and promotes substrate degradation
Source: Sci Adv. 2022 Dec 23;8(51):eadd9520. doi: 10.1126/sciadv.add9520 (PMC9788759; doi:10.1126/sciadv.add9520)
Supplement: Supplementary file 1 — Figs. S1 to S17 Tables S1 to S5 [file sciadv.add9520_sm.pdf]

Science Advances
AAAS

Supplementary Materials for

Ubiquitin modulates 26*S* proteasome conformational dynamics and promotes substrate degradation

Erik Jonsson *et al.*

Corresponding author: Andreas Martin, a.martin@berkeley.edu

Sci. Adv. **8**, eadd9520 (2022)
DOI: 10.1126/sciadv.add9520

This PDF file includes:

Figs. S1 to S17
Tables S1 to S5

Supplementary Figures:

a Heterologous expression and reconstitution system

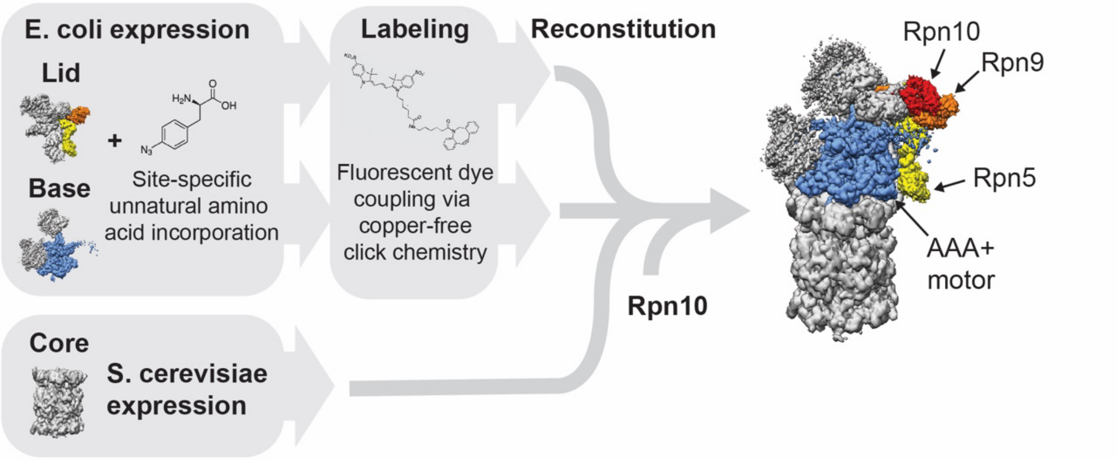

	Donor (D) position	Acceptor (A) position	Proteasome conformation	PDB ID	Cα distance between D and A attachment points (Å)
Substrate-processing assay	I27-tail C127	Rpt1 I191AzF	non-s1 (substrate engaged)	6EF3	23 *
Conformational change assay	Rpn9 F2AzF	Rpt5 Q49AzF	s1	5MP9	77 #
	Rpn9 F2AzF	Rpt5 Q49AzF	non-s1 (s3)	5MPB	41 #

b Substrate-protein design

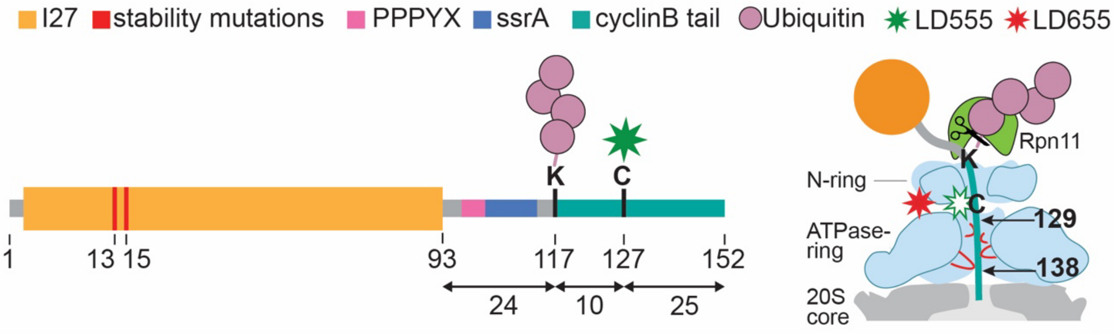

I27 substrate sequence	MGGGLIEVERPLYGVEVFVGETAHFEIELSEPDVHGQWRLRGQPLAASPDCEIIEDGRRHILILHNCQLGMTGEVSFQAANTRSAANLRVRELGGAGPPPYSAANDENYALAAHGG**K**HTFNNENVS**C**RLGGAASIAVQAPAQHTFNNENVSY
I27 substrate with glycine-serine-rich-tail sequence (I27-GS)	MGGGLIEVERPLYGVEVFVGETAHFEIELSEPDVHGQWRLRGQPLAASPDCEIIEDGRRHILILHNCQLGMTGEVSFQAANTRSAANLRVRELGGAGPPPYSAANDENYALAAHGG**K**HTFNNENVS**C**QAPAQHTFNNENVSSGSSSSSSASY

Figure S1: Heterologous proteasome expression and substrate-protein design. a) Proteasome lid and base subcomplexes from *Saccharomyces cerevisiae* are heterologously expressed in *E. coli*, site-specifically labeled through incorporation of the unnatural amino acid azido-phenylalanine (AzF) and copper-free click addition of fluorescent dyes, and reconstituted with the 20S core particle purified from *S. cerevisiae*. The table lists the labeling positions, the considered proteasome structures and conformational states, and the Cα distances between labeled residues for the FRET-based substrate-processing and conformational-change assays. * The Cα distance for the substrate processing assay is given for the fully inserted substrate tail as observed in the substrate-engaged proteasome structure immediately prior to deubiquitination [35]. # The Cα position for F2 in Rpn9 was determined based on the proteasome structures 5MP9 and 5MPB in combination with the 2MR3 structure of Rpn9 in solution. **b**) Substrate proteins include the titin I27 domain (yellow) from which all lysines have been removed and a C-terminally fused unstructured initiation region or "tail" derived from a Cyclin-B sequence (teal) for optimal substrate engagement (I27 substrate sequence). For partially impaired engagement by the proteasome we also generated an I27 substrate with glycine-serine-rich tail (I27-GS), in which the Cyclin-B sequence is followed by a 10-residue glycine/serine-rich region (purple). In both substrate designs, the tail contains a single lysine and PPPY motif (pink) for Rsp5-catalyzed polyubiquitination, a single cysteine for maleimide labeling with a donor fluorophore (LD555 or Cy3), and a ssrA sequence (blue) for binding to proteasome-fused SspB in the ubiquitin-independent delivery system. A N-terminal GGG motif is used to attach fluorescein (FAM) in a sortase-catalyzed reaction for substrate-degradation measurements in bulk. The thermodynamic stability of the I27 domain can be modulated by the incorporation of the V13P and V15P point mutations (red). The schematic on the right is based on our previous cryo-EM structure [35] and shows the substrate-engaged state of the proteasome prior to deubiquitination (PDB ID: 6EF3). For the substrate-processing assay, dye positions on the substrate and the proteasome were chosen to give a high-FRET signal in this engaged state after complete tail insertion, in which ~ 138 residues of the I27 model substrate reside above or inside the central channel of the motor.

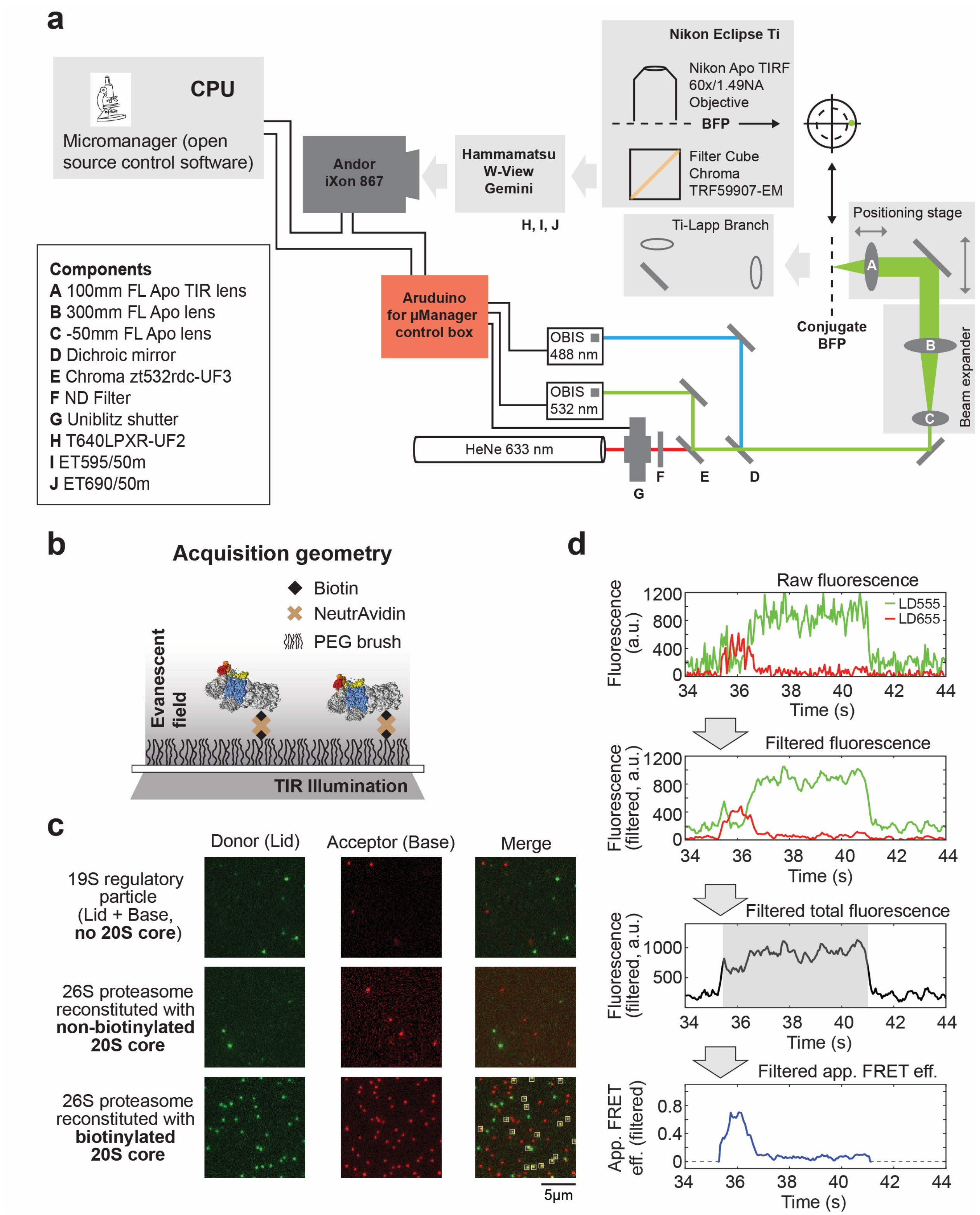

Figure S2: Single-molecule total internal reflection fluorescence (TIRF) microscope design and assay setup. a) Diagram of the custom-built TIRF microscope used in this study. **b)** Cartoon

representation depicting the assay geometry. Singly labeled proteasomes for the substrate-processing assay or doubly labeled proteasomes for the conformational-change assay were immobilized to the surface of a PEGylated cover glass through biotin-NeutrAvidin interaction. **c)** Raw microscopy images of the donor (LD555)-labeled lid (green), the acceptor (LD655)-labeled base (red), and the overlay are shown for the 19S regulatory particle (top), the 26S proteasome reconstituted with non-biotinylated, unlabeled 20S core (middle) and the 26S proteasome reconstituted with biotinylated, unlabeled 20S core (bottom). Scale bar represents 5μm. The colocalized donor and acceptor fluorescent spots (white boxes) were only observed in the 26S proteasome reconstituted with biotinylated core particle. **d)** Data-analysis workflow for the substrate processing assay monitored by 532-nm laser excitation. Traces for raw donor fluorescence and raw acceptor fluorescence are extracted from the spatially aligned microscope images and filtered using MATLAB's smooth function with a heuristically determined averaging window of 5 frames (equivalent to 250 ms). Total fluorescence (sum of donor and acceptor fluorescence signals) with a threshold 2-fold above background is used to determine substrate-degradation events (indicated by grey shading), which start with the binding of the donor-labeled substrate to the proteasome and end with the release of donor-labeled peptide products. The apparent FRET efficiency outside these degradation events is not determined and indicated by a black dashed line. The filtered FRET-efficiency trace is used to analyze the kinetics of individual processing steps as described in methods.

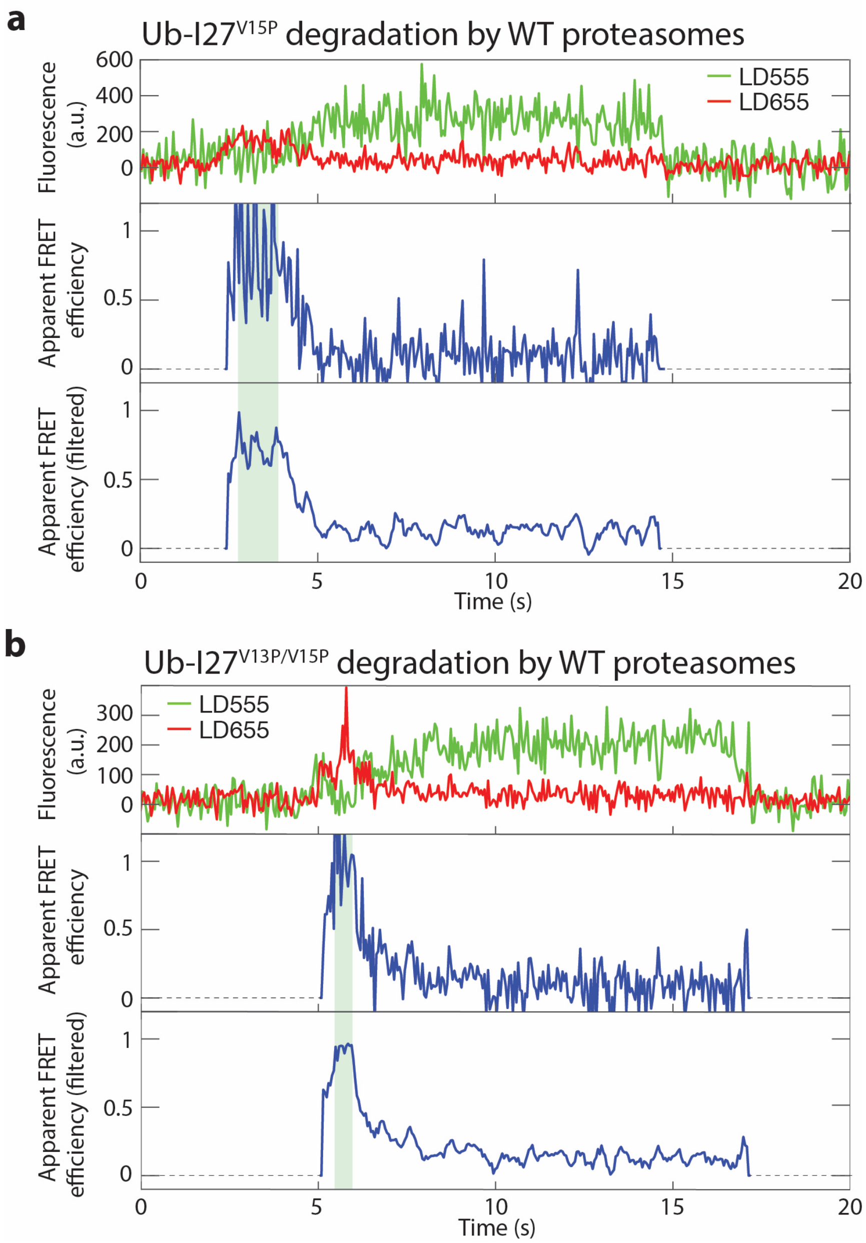

Figure S3: Representative traces for the substrate-processing assay. Example traces for the degradation of **a)** ubiquitinated I27^{V15P} substrate and **b)** ubiquitinated I27$^{V13P/V15P}$ substrate by wild-type proteasome monitored with 532-nm laser excitation. Raw donor (LD555, green) and acceptor (LD655, red) fluorescence traces are shown in the top panels, the corresponding traces for raw apparent FRET efficiencies in the middle, and the filtered apparent FRET efficiencies in the bottom panels. FRET efficiencies outside of degradation events are not defined and represented by dotted lines. The green shading indicates the high-FRET deubiquitination phase.

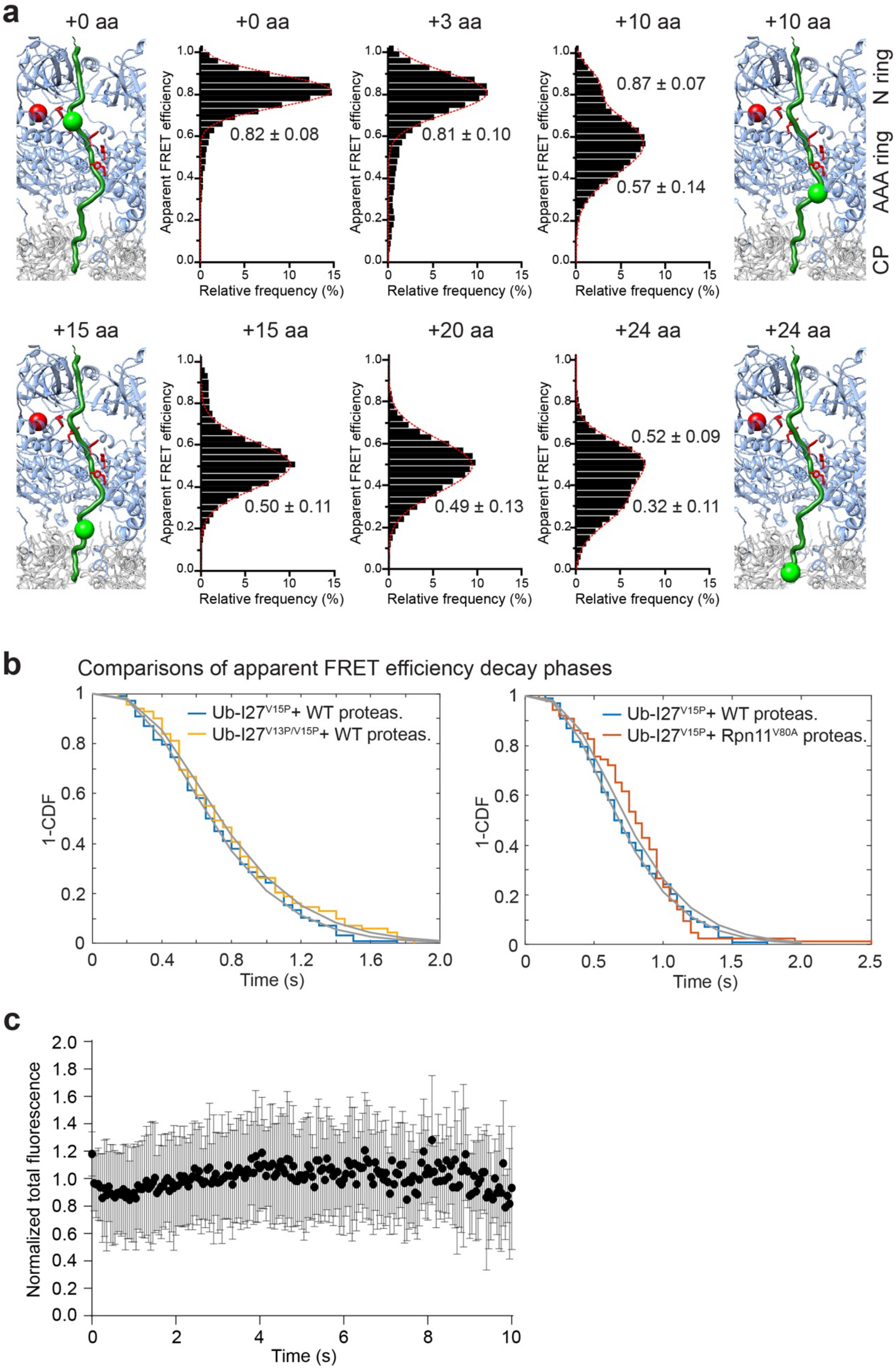
a
+0 aa
+3 aa
+10 aa
+15 aa
+20 aa
+24 aa
N ring
AAA ring
CP
Apparent FRET efficiency
Relative frequency (%)
0.82 ± 0.08
0.81 ± 0.10
0.87 ± 0.07
0.57 ± 0.14
0.50 ± 0.11
0.49 ± 0.13
0.52 ± 0.09
0.32 ± 0.11
b
Comparisons of apparent FRET efficiency decay phases
Ub-I27^V15P+ WT proteas.
Ub-I27^V13P/V15P+ WT proteas.
Ub-I27^V15P+ Rpn11^V80A proteas.
1-CDF
Time (s)
c
Normalized total fluorescence
Time (s)

Figure S4: Analysis of the FRET-efficiency decay phase in the substrate processing assay. a) Translocation reference to correlate changes in apparent FRET efficiency to substrate movement through the central channel. Shown are the histograms of the apparent FRET-efficiency distributions for different ubiquitinated I27^{V15P} substrate variants that were stalled in the ATPase motor to approximate the number of amino acids translocated during the FRET-decay phase of substrate-processing traces. The cysteine residue used for donor (LD555) labeling is moved from the original position (+ 0 aa), where the highest apparent FRET efficiency is observed after complete tail insertion, further towards the C-terminus of the substrate tail region and thus downwards in the central channel by increments of up to 24 residues. To stall the substrate after complete tail insertion, the catalytically dead Rpn11^{AXA}-lid was used for the proteasome reconstitution and the imaging buffer was supplemented with ATPγS. Fit lines in red and the average FRET-efficiency values next to them were derived by fitting a Gaussian distribution or the sum of two Gaussian distributions to the histograms. Bimodal distributions likely indicate alternative orientations of the bulky donor dye within the narrow channel of the ATPase motor. The structural representations on the far left and right are based on our cryo-EM structure of the 26S proteasome bound to a stalled substrate (PDB ID: 6EF3), with the base ATPase motor (N ring and AAA ring) in blue, the 20S core particle (CP) in gray, the pore-1 loop tyrosines in red stick representation, and the substrate in green. Rpt4 and the alpha-7 subunit of CP are hidden for a clearer view of the substrate bound in the central channel. The Cα atom of I191 in Rpt1 as the acceptor-dye attachment point (I191AzF) is shown as a red sphere, and the Cα atoms of engineered cysteines at the +0, +10, +15, and +24 positions as the donor-dye attachment points in the stalled substrate are depicted as green spheres. **b)** Left, survival plots of the length of the FRET-efficiency decay phase after deubiquitination during the degradation of ubiquitinated I27^{V15P} (blue, N = 98) and I27$^{V13P/V15P}$ substrates (orange, N = 69) by wild-type proteasome. The survival plots were compared using the Mantel-Cox test, yielding a p-value of 0.2411. Right, survival plots of the length of the FRET-efficiency decay phase after deubiquitination for the degradation of ubiquitinated I27^{V15P} by wild-type proteasome (blue, N = 98) and Rpn11^{V80A}-mutant proteasome (red, N = 86). The survival plots were compared using the Mantel-Cox test, yielding a p-value of 0.5754. **c)** Averages of the normalized total fluorescence values (sum of

donor and acceptor fluorescence, normalized based on the first 5 frames for each trace) during individual degradation events that were monitored by the FRET-based substrate-processing assay (LD555 on the substrate and LD655 on Rpt1) and temporally aligned. Error bars depict the standard deviation (N = 50). The total fluorescence does not show major changes that could be indicative of significant protein-induced fluorescence enhancement (PIFE) of the substrate-attached donor dye during its passage through the proteasome central channel.

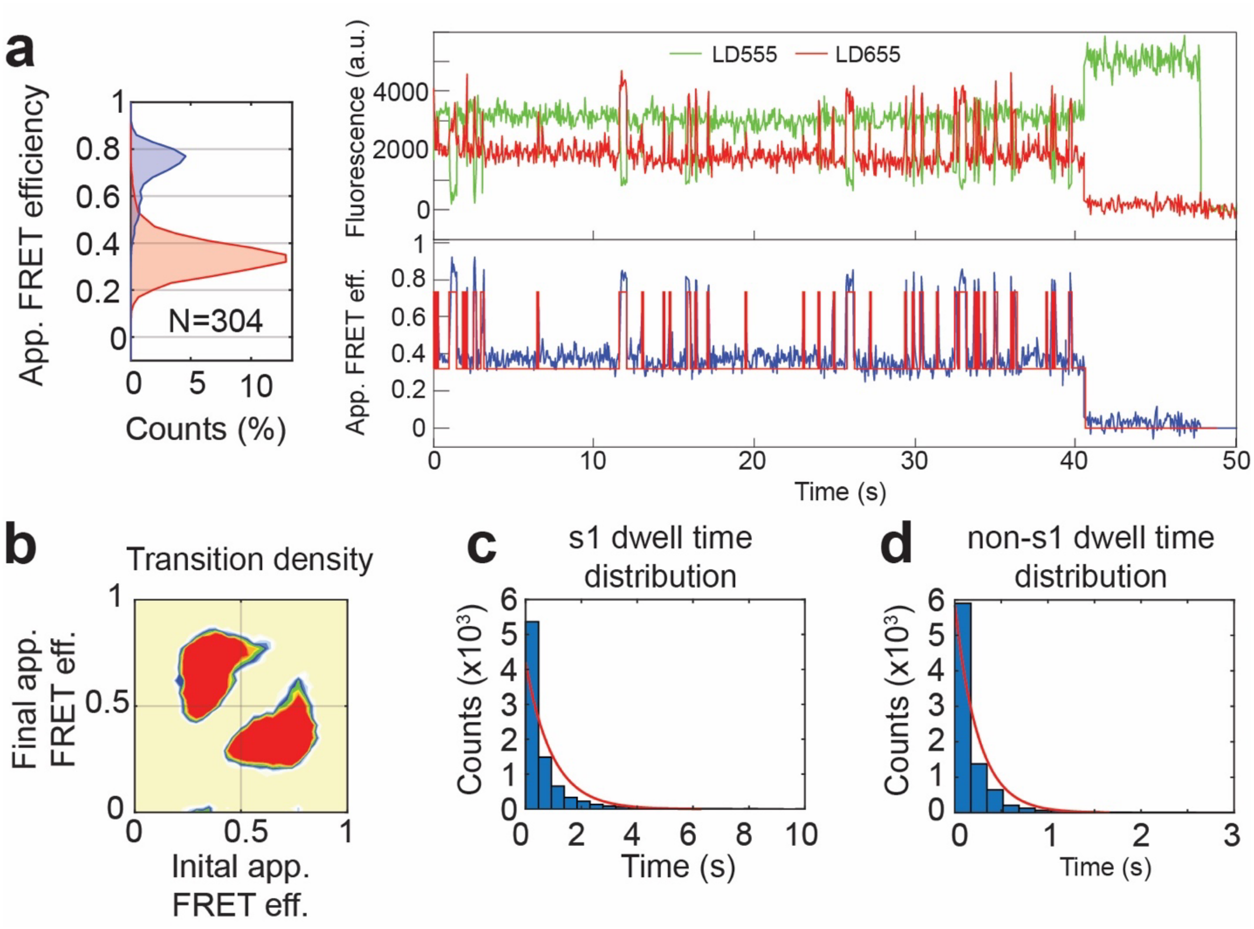

Figure S5: Hidden Markov modeling of conformational switching. a) Representative raw donor (LD555, green) and acceptor (LD655, red) fluorescence traces (top panel), and the apparent FRET efficiency trace (blue) of the conformational-change assay for wild-type proteasome in ATP monitored through 532-nm laser excitation, with dynamic switching fit according to a two-state system (solid red line). The histogram on the left for multiple traces shows the relative occupancy of each state. **b)** Transition density plot depicting the apparent FRET efficiency values before and after each transition. **c)** Dwell-time distribution for the low-FRET s1 state is fit to a single exponential (red line) from which transition rates can be derived. **d**) Dwell-time distribution for the high-FRET non-s1 states is fit to a single exponential (red line).

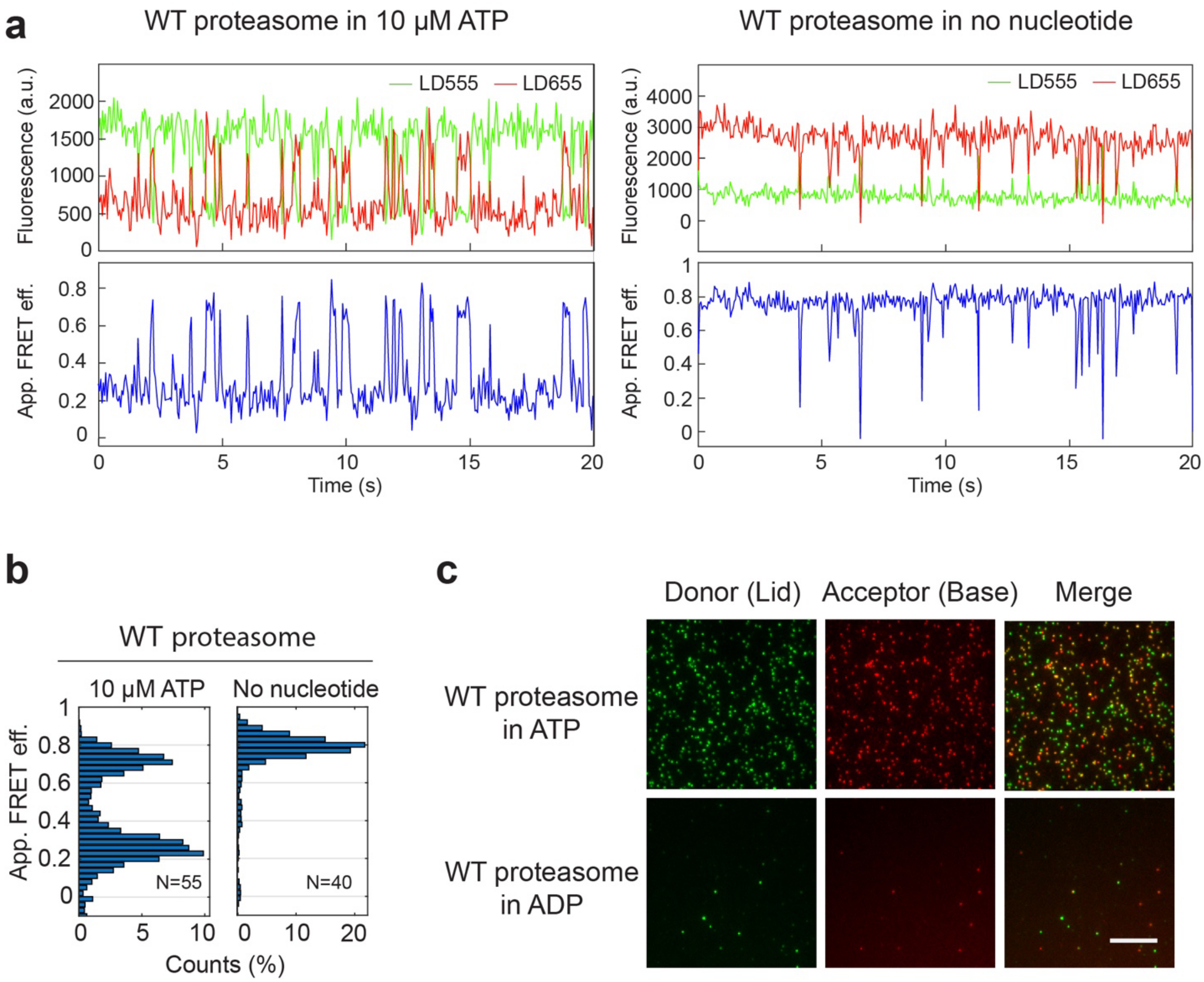

Figure S6: Nucleotide dependence of proteasome conformational dynamics. a) Representative trace of wild-type proteasome in 10 μM ATP (left) exhibits more frequent and longer dwells for non-s1 states than at saturating ATP concentrations (c.f. Fig. 2b). Representative trace of wild-type proteasome in the absence of ATP (right) shows dominant non-s1 states with rare, brief transitions to the s1 state. Raw donor (LD555, green) and acceptor (LD655, red) fluorescence traces monitored after 532-nm laser excitation are shown in the top panel and the corresponding apparent FRET efficiency trace (blue) is shown in the bottom panel. **b**) Histograms for wild-type proteasomes at low ATP concentrations and without ATP show a bias towards the high-FRET non-s1 states. **c)** ADP promotes the disassembly of the proteasome holoenzyme. Raw TIRF fluorescence images (excitation at 532 nm) of reconstituted proteasomes with donor-labeled lid (green), acceptor-labeled base (red), and unlabeled core particle show stable association at

saturating ATP concentrations (top), but dissociation after buffer exchange with ADP (bottom). Scale bar represents 10 µm.

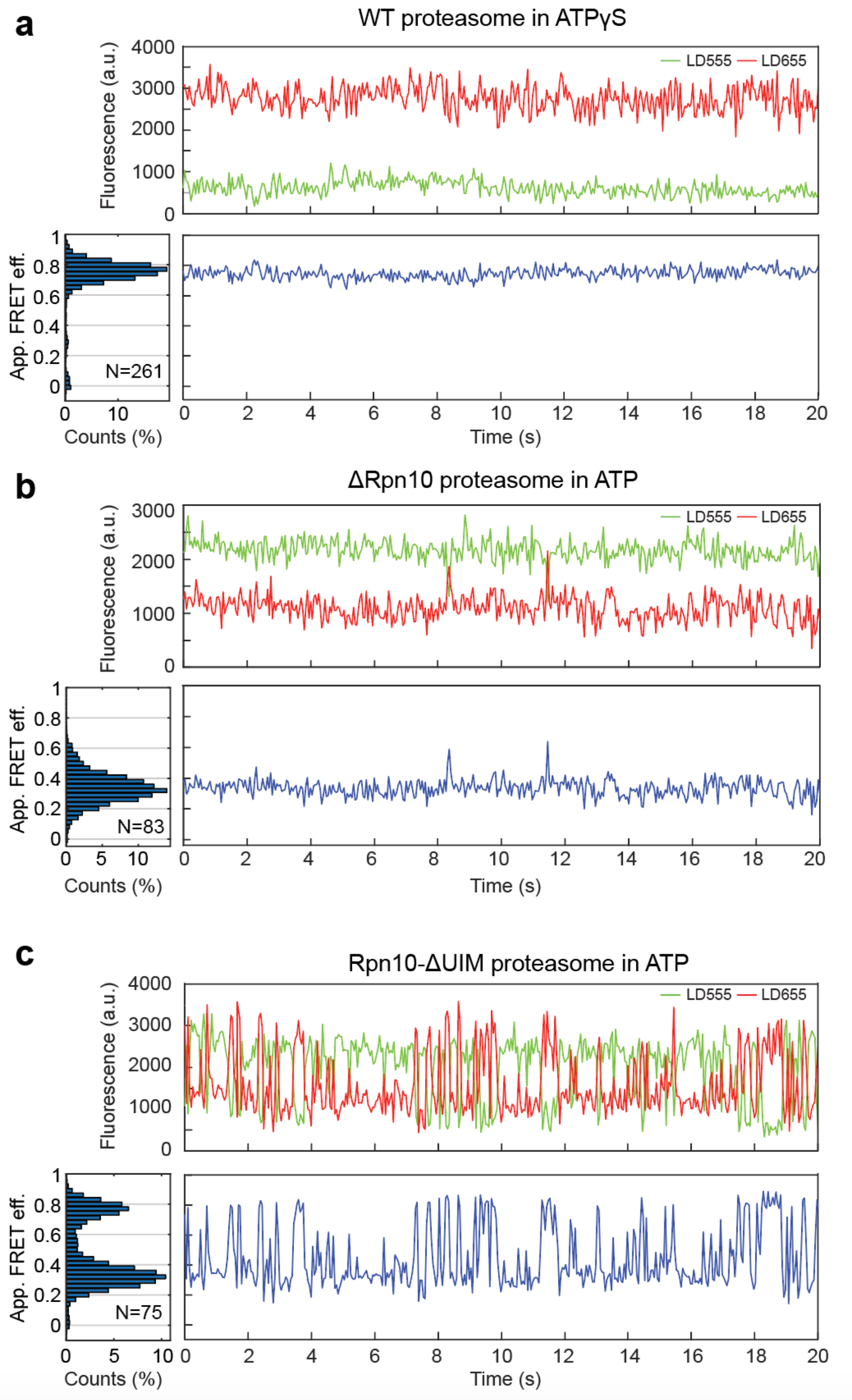

a
WT proteasome in ATPγS
LD555
LD655
Fluorescence (a.u.)
App. FRET eff.
N=261
Counts (%)
Time (s)
b
ΔRpn10 proteasome in ATP
LD555
LD655
Fluorescence (a.u.)
App. FRET eff.
N=83
Counts (%)
Time (s)
c
Rpn10-ΔUIM proteasome in ATP
LD555
LD655
Fluorescence (a.u.)
App. FRET eff.
N=75
Counts (%)
Time (s)

d

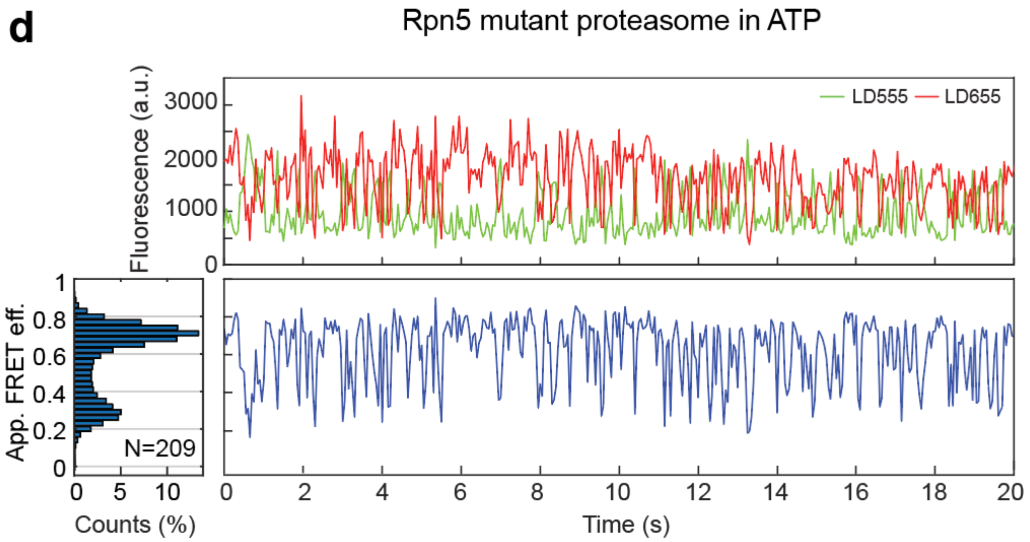

Figure S7: Representative conformational dynamics traces for proteasome under various conditions. Raw donor (LD555, green) and acceptor (LD655, red) fluorescence traces monitored after 532-nm laser excitation are shown in the top panels and the corresponding apparent FRET efficiency trace (blue) is shown in the bottom panels. **a)** Example trace for the conformational changes of wild-type proteasome in ATPγS exhibits a stable high-FRET, non-s1 state. The histogram on the left indicates that the entire population is similarly biased towards non-s1 states. **b)** Example trace for the ΔRpn10 proteasome shows almost complete conformational bias towards the low FRET s1 state. A histogram for a population of ΔRpn10 proteasomes is depicted on the left. **c)** Representative trace for Rpn10-ΔUIM proteasomes exhibits longer dwells in high-FRET non-s1 states, consistent with the UIM stabilizing the s1 state (c.f. Table S2). The population histogram (left) for this mutant proteasome exhibits a more pronounced high-FRET compared to the wild-type proteasome (c.f. Figure 2b). **d)** Representative trace and histogram for Rpn5-mutant holoenzymes exhibits frequent switching and bias towards the high-FRET non-s1 states.

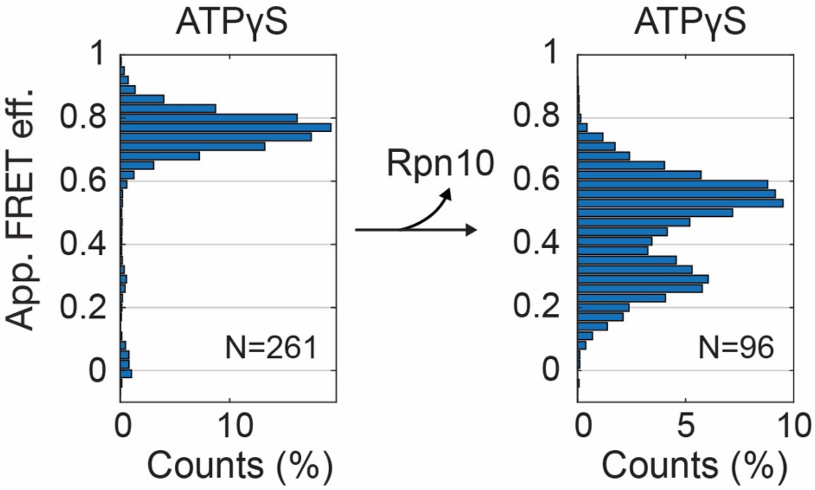

Figure S8: Rpn10 stabilizes the non-s1 state. Histograms for the conformational equilibria of wild-type (left) and ΔRpn10 proteasomes (right) in ATPγS show a shift from a dominant non-s1 population to a bimodal distribution upon Rpn10 deletion. Non-s1 states of the ΔRpn10 proteasome show slightly lower apparent FRET efficiency values (~ 0.6 versus ~ 0.8 for wild type), possibly due to a reorientation of the Rpt5-attached acceptor dye.

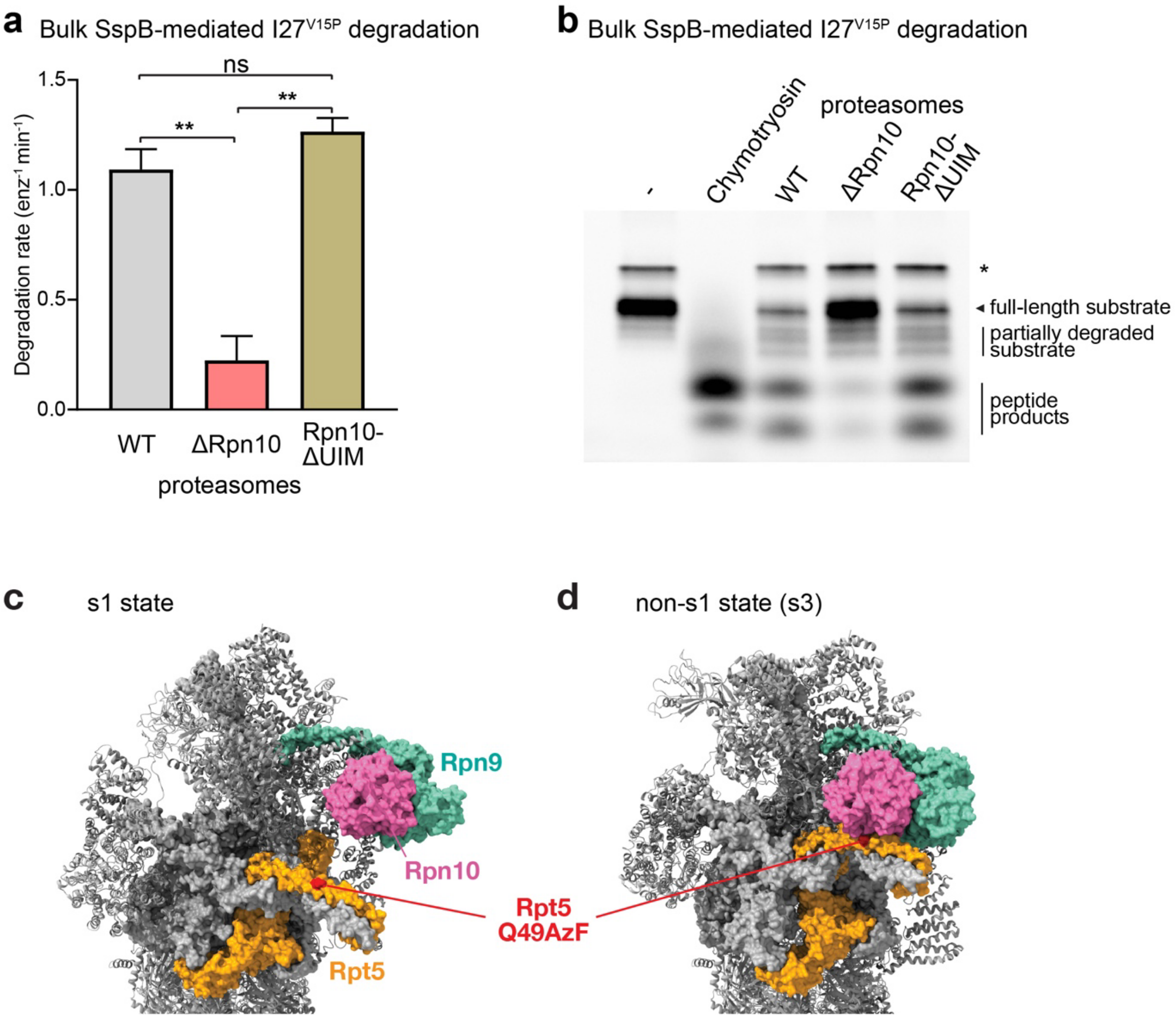

Figure S9: Dissecting the role of Rpn10 in substrate degradation using the SspB-mediated ubiquitin-independent delivery system. a) Rates for the multiple-turnover degradation of the $I27^{V15P}$ substrate after SspB-mediated delivery to wild-type (grey), ΔRpn10 (pink), or Rpn10-ΔUIM (gold) proteasomes (N = 3 technical replicates). Statistical significance was calculated using an unpaired two-tailed Welch's t-test: ** $p < 0.005$; ns, $p = 0.2053$. **b)** FAM-fluorescence detection after SDS PAGE reveals similar peptide products for the degradation of the FAM-labeled $I27^{V15P}$ substrate by SspB-fused wild-type, ΔRpn10, or Rpn10-ΔUIM proteasomes. Chymotryptic cleavage of the substrate was used as a positive control for peptide formation. * indicates a contamination with FAM-labeled sortase. **c-d)** Structures of the proteasome in the s1 state (c, PDB ID: 6FVT) and the s3 state (d, PDB ID: 6FVV), with the base ATPase subunits shown in grey surface representation, except for Rpt5, which is shown in orange. Rpn10's VWA domain is shown in pink

and Rpn9 in teal. While in the s1 state Rpn10 is spatially separated from the Rpt5 N-terminal coiled coil, it makes direct contact in the non-s1 (s3) states, which likely explains why its deletion leads to a potential reorientation of the Rpt5-attached dye and a consequent lower FRET value for non-s1 states of ΔRpn10 proteasomes.

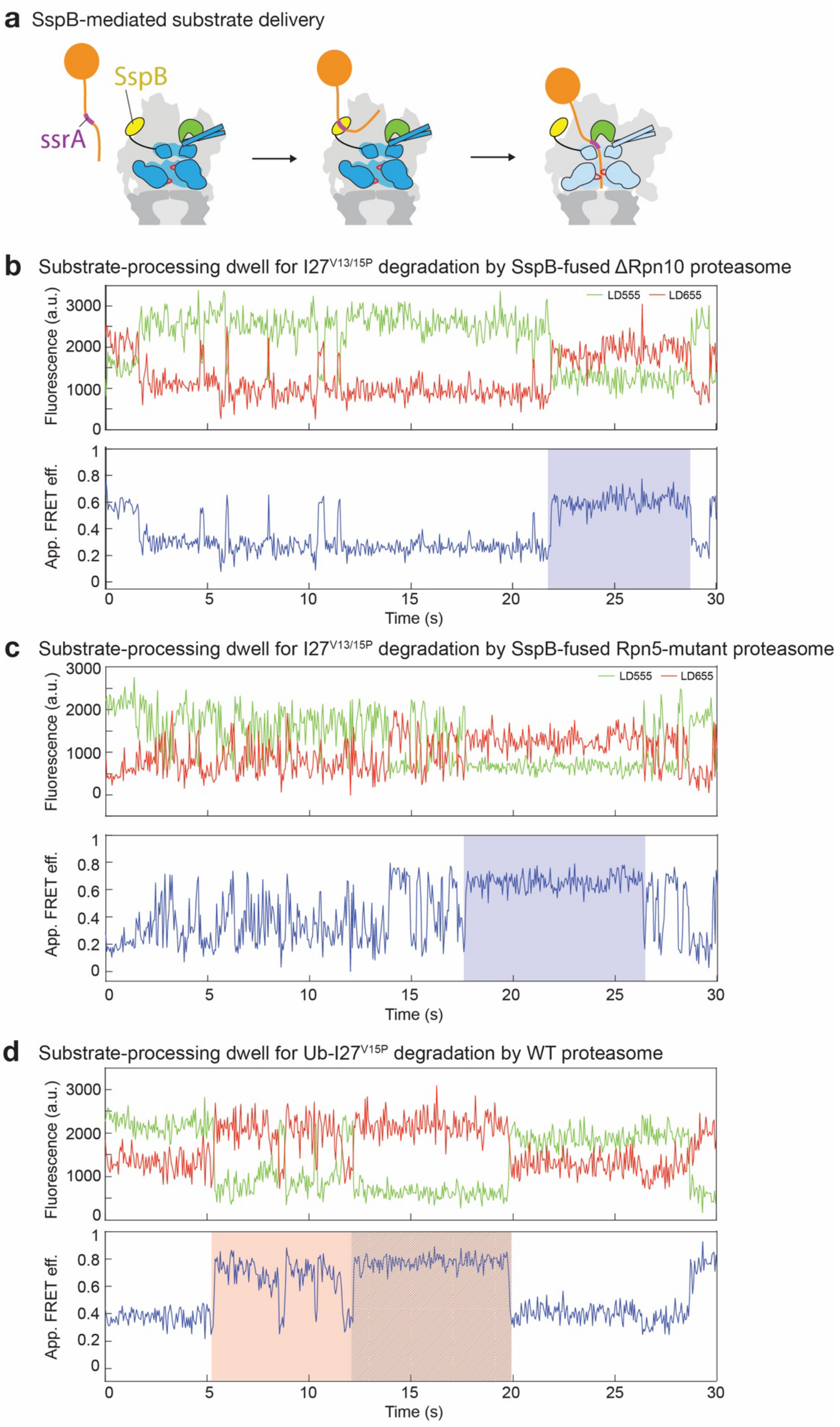
a SspB-mediated substrate delivery
SspB
ssrA
b Substrate-processing dwell for I27V13/15P degradation by SspB-fused ΔRpn10 proteasome
Fluorescence (a.u.)
LD555
LD655
App. FRET eff.
Time (s)
c Substrate-processing dwell for I27V13/15P degradation by SspB-fused Rpn5-mutant proteasome
Fluorescence (a.u.)
LD555
LD655
App. FRET eff.
Time (s)
d Substrate-processing dwell for Ub-I27V15P degradation by WT proteasome
Fluorescence (a.u.)
App. FRET eff.
Time (s)

Figure S10: Representative traces for substrate-induced transitions to non-s1 states under various conditions. a) Schematic for ubiquitin-independent substrate delivery through the interaction between the proteasome-fused SspB (yellow) and the ssrA sequence (magenta) in the unstructured tail of the substrate. **b)** Representative trace for the conformational change assay during $I27^{V13/15P}$ substrate degradation by SspB-fused ΔRpn10 proteasome. Raw donor (LD555, green) and acceptor (LD655, red) fluorescence traces monitored after 532-nm laser excitation are shown in the top panel and the corresponding FRET trace (blue) is shown in the bottom panel. The processing dwells (blue shading) for this substrate are characterized by their lack of transitions from the high-FRET non-s1 states to the low-FRET s1 state. **c)** Representative trace for the conformational change assay during $I27^{V13/15P}$ substrate degradation by the SspB-fused Rpn5-mutant proteasome shows a substrate-processing dwell that also lacks s1 excursions. **d)** Representative trace for the conformational change assay during ubiquitin-dependent degradation of the $I27^{V15P}$ substrate. The substrate-processing dwell (red shading) shows brief excursions to the low-FRET s1 state during the unfolding phase, but excursions are absent during translocation (hatched; see also Fig. 3a, Fig. S11, S16).

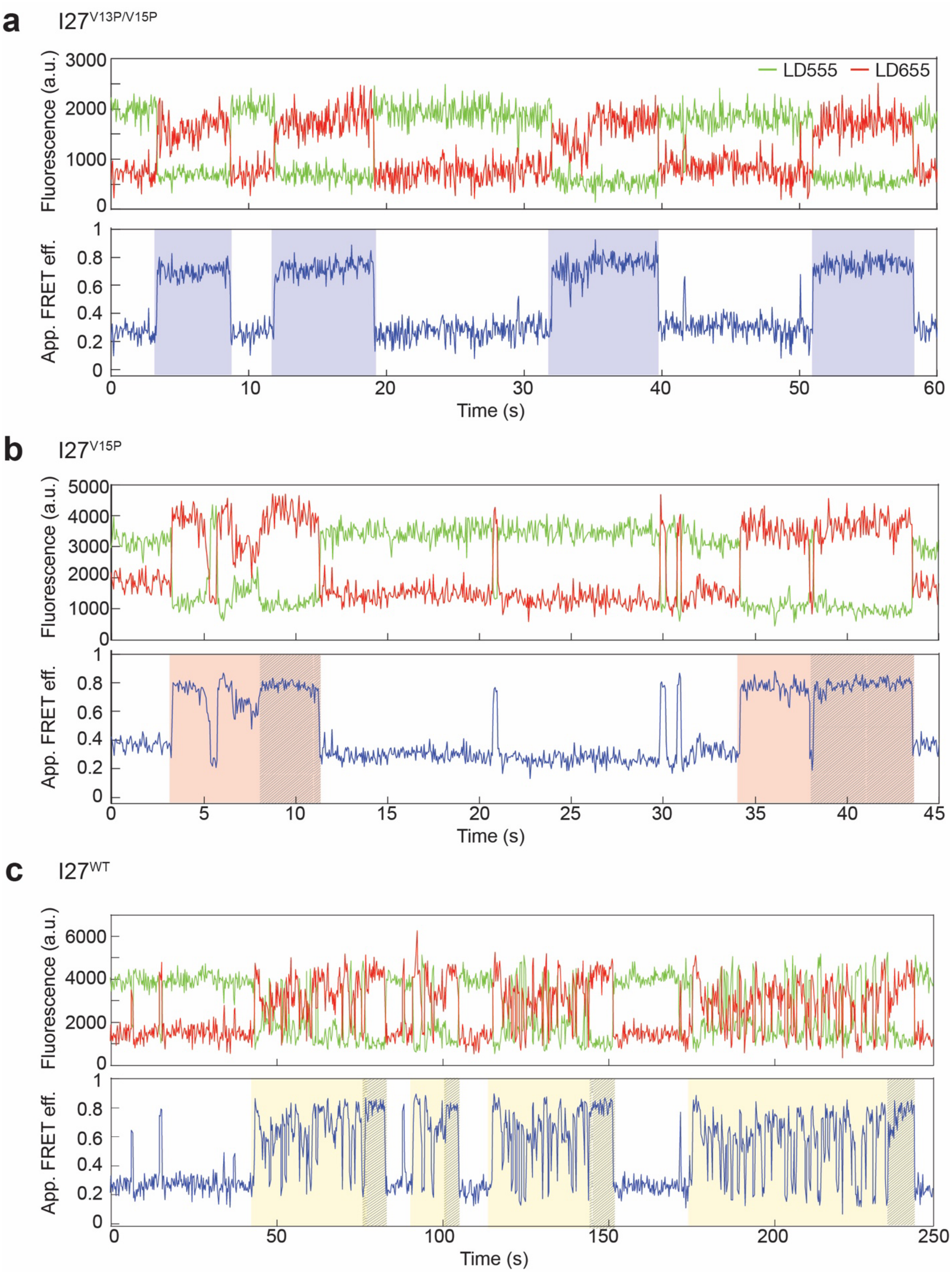
a
I27V13P/V15P
LD555
LD655
Fluorescence (a.u.)
App. FRET eff.
Time (s)
b
I27V15P
Fluorescence (a.u.)
App. FRET eff.
Time (s)
c
I27WT
Fluorescence (a.u.)
App. FRET eff.
Time (s)

Figure S11: Substrate processing dwells and s1-state excursions. Raw donor (LD555, green) and acceptor (LD655, red) fluorescence traces monitored after 532-nm laser excitation are shown in the top panels and the corresponding apparent FRET efficiency trace (blue) is shown in the bottom panels. **a)** Representative trace for the conformational change assay with four consecutive substrate processing events (blue shading) for SspB-mediated degradation of $I27^{V13/15P}$ substrate. Dwells for this thermodynamically labile substrate do not exhibit pronounced transitions from the high-FRET non-s1 states to the low-FRET s1 state. **b)** Representative trace with two consecutive substrate-processing events (red shading) for SspB-mediated degradation of $I27^{V15P}$ substrate. Dwells for this substrate show s1 transitions early, potentially during unsuccessful unfolding attempts, but lack them during the last 3-5 s (hatched), which likely represents translocation of the unfolded polypeptide. **c)** Representative trace with four consecutive substrate-processing events (yellow shading) for SspB-mediated degradation of wild-type I27 substrate. Dwells for this thermodynamically most stable substrate exhibit frequent excursions to the low-FRET s1 state, except for the last 3-5 s (hatched).

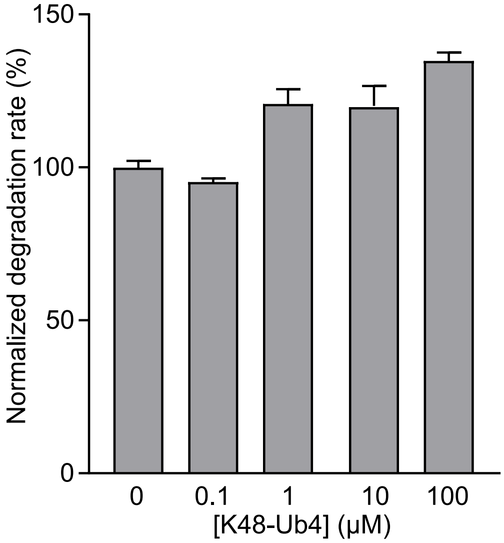

Figure S12: Unanchored K48-linked tetra-ubiquitin chains accelerate the multiple turn-over degradation rates of $I27^{V15P}$ substrate in a concentration-dependent manner. Degradation rates were normalized to 100% in the absence of unanchored K48-linked tetra-ubiquitin chains.

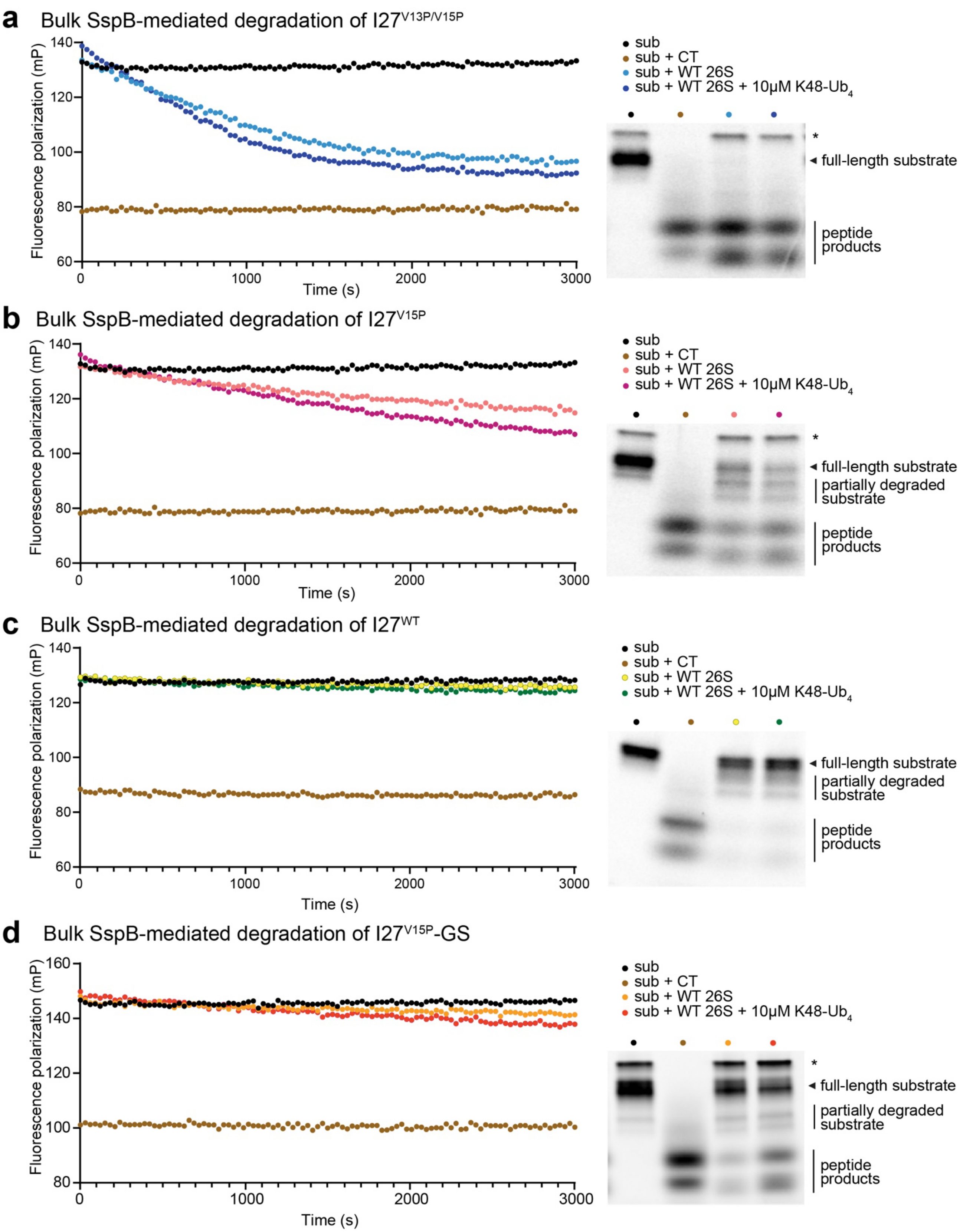
a
Bulk SspB-mediated degradation of I27V13P/V15P
Fluorescence polarization (mP)
140
120
100
80
60
0
1000
2000
3000
Time (s)
sub
sub + CT
sub + WT 26S
sub + WT 26S + 10μM K48-Ub4
*
full-length substrate
peptide products
b
Bulk SspB-mediated degradation of I27V15P
Fluorescence polarization (mP)
140
120
100
80
60
0
1000
2000
3000
Time (s)
sub
sub + CT
sub + WT 26S
sub + WT 26S + 10μM K48-Ub4
*
full-length substrate
partially degraded substrate
peptide products
c
Bulk SspB-mediated degradation of I27WT
Fluorescence polarization (mP)
140
120
100
80
60
0
1000
2000
3000
Time (s)
sub
sub + CT
sub + WT 26S
sub + WT 26S + 10μM K48-Ub4
full-length substrate
partially degraded substrate
peptide products
d
Bulk SspB-mediated degradation of I27V15P-GS
Fluorescence polarization (mP)
160
140
120
100
80
0
1000
2000
3000
Time (s)
sub
sub + CT
sub + WT 26S
sub + WT 26S + 10μM K48-Ub4
*
full-length substrate
partially degraded substrate
peptide products

Figure S13: Representative fluorescence polarization data for multiple-turnover degradation of various model substrates in the SspB-delivery system. For each FAM-labeled I27-substrate variant, the fluorescence polarization of the substrate alone is depicted in black and that of substrate digested with chymotrypsin (CT) in brown. SDS-PAGE gels are shown for visualization of the degradation products, * indicates a contamination with FAM-labeled sortase. **a)** Representative fluorescence polarization trace of multiple-turnover degradation of $I27^{V13P/V15P}$ by wild-type proteasome (WT 26S) in the absence (light blue) and presence (dark blue) of unanchored K48-linked tetraubiquitin chains ($K48\text{-}Ub_4$). **b)** Representative fluorescence polarization trace of multiple-turnover degradation of $I27^{V15P}$ in the absence (light orange) and presence (pink) of unanchored $K48\text{-}Ub_4$. **c)** Representative fluorescence polarization trace of multiple-turnover degradation of $I27^{WT}$ in the absence (yellow) and presence (green) of unanchored $K48\text{-}Ub_4$. **d)** Representative fluorescence polarization trace of multiple-turnover degradation of the $I27^{V15P}$-GS substrate with Gly/Ser-rich tail (see Fig. S1b) in the absence (light orange) and presence (red) of unanchored $K48\text{-}Ub_4$.

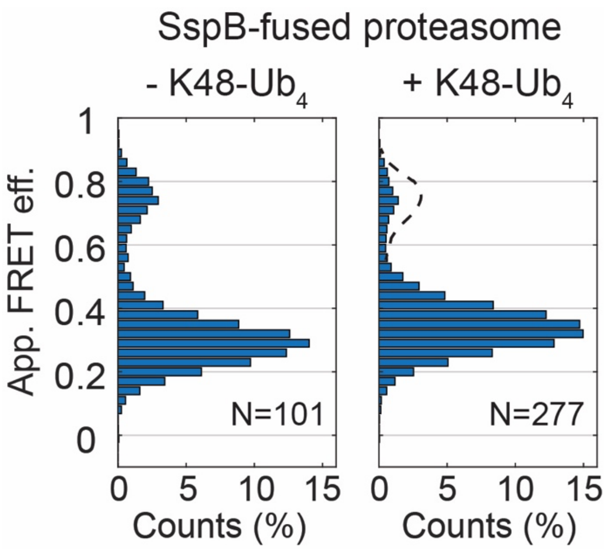

Figure S14: Ubiquitin chains modulate the conformational dynamics of the SspB-fused proteasome similar to wild-type proteasome. FRET-state histograms for SspB-fused proteasomes in the absence (left) and presence (right) of K48-linked tetra-ubiquitin chains (K48-Ub_4).

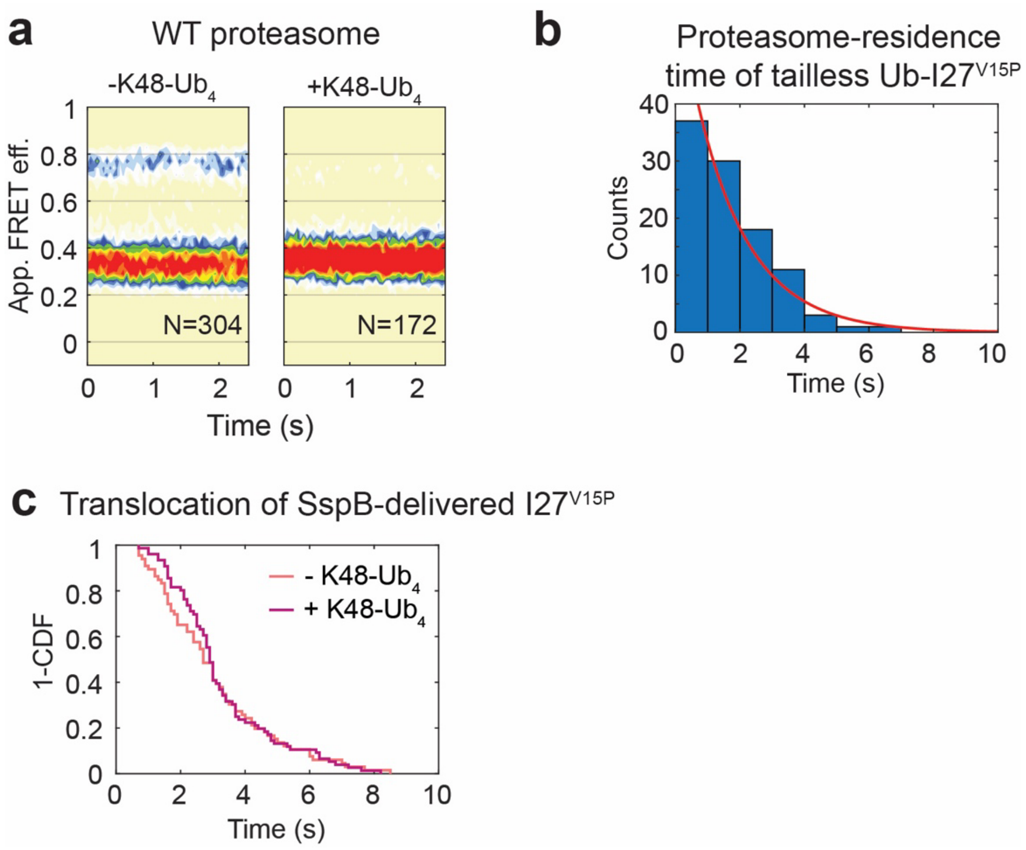

Figure S15: Proteasome interactions with ubiquitin. a) Contour plots for the FRET-state occupancies of wild-type proteasome in the absence (left) and presence (right) of unanchored K48-Ub_4. This is an alternative representation of the data shown in figure 3b. **b)** Dwell-time histogram for the residence time of a ubiquitinated tailless $I27^{V15P}$ substrate on the proteasome (N = 101). This substrate is unable to engage with the ATPase motor and therefore only exhibits non-productive binding, indicated by short events with intermediate FRET value (see figure 4d). The histogram was fit to a single exponential (red line) to determine the time constant τ_{off} = 0.61 ± 0.12 s for the dissociation of an ubiquitinated substrate from proteasomal receptors prior to engagement (error is 95% confidence interval of the fit). **c)** FRET-decay times in the substrate-processing assay for the degradation of $I27^{V15P}$ show no difference in the absence versus presence of K48-Ub_4 (N = 66 and 76 respectively). The survival plots were compared using the Mantel-Cox test, with a p-value of 0.7682.

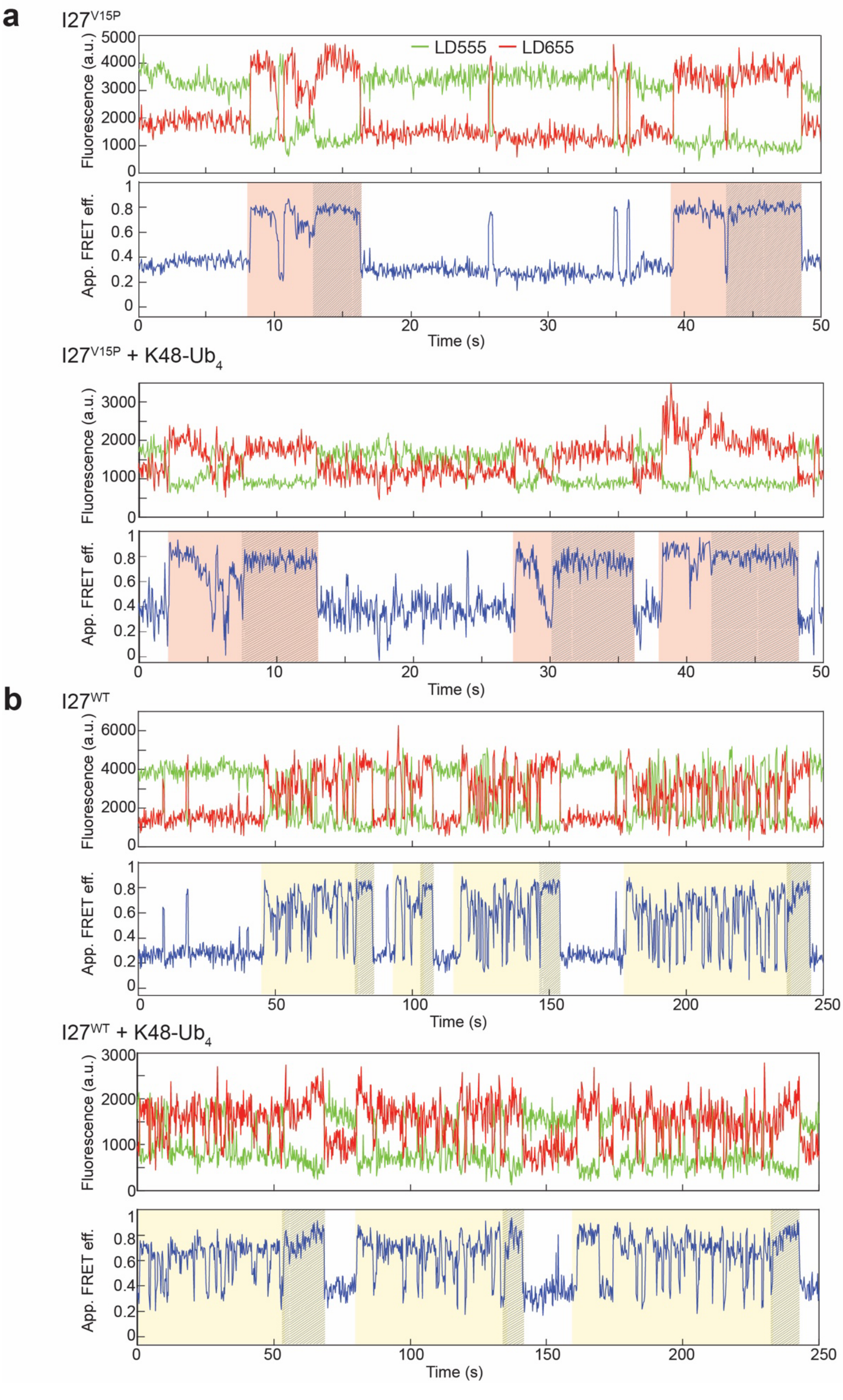
a
I27V15P
LD555
LD655
Fluorescence (a.u.)
App. FRET eff.
Time (s)
I27V15P + K48-Ub4
b
I27WT
I27WT + K48-Ub4

Figure S16: No obvious modulation of proteasome conformational dynamics by ubiquitin during substrate degradation. Raw donor (LD555, green) and acceptor (LD655, red) fluorescence traces monitored via 532nm laser excitation are shown in the top panels and the corresponding apparent FRET efficiency trace (blue) is shown in the bottom panels. **a)** Representative traces for the conformational change assay monitoring SspB-mediated $I27^{V15P}$ degradation in the absence and presence of K48-Ub_4 show no significant difference in the frequency or duration of excursions to the low-FRET s1 state during substrate-processing dwells. **b)** Similarly, processing dwells during the SspB-mediated $I27^{WT}$ degradation exhibit no discernable differences in conformational switching behavior between the absence and presence of K48-Ub_4.

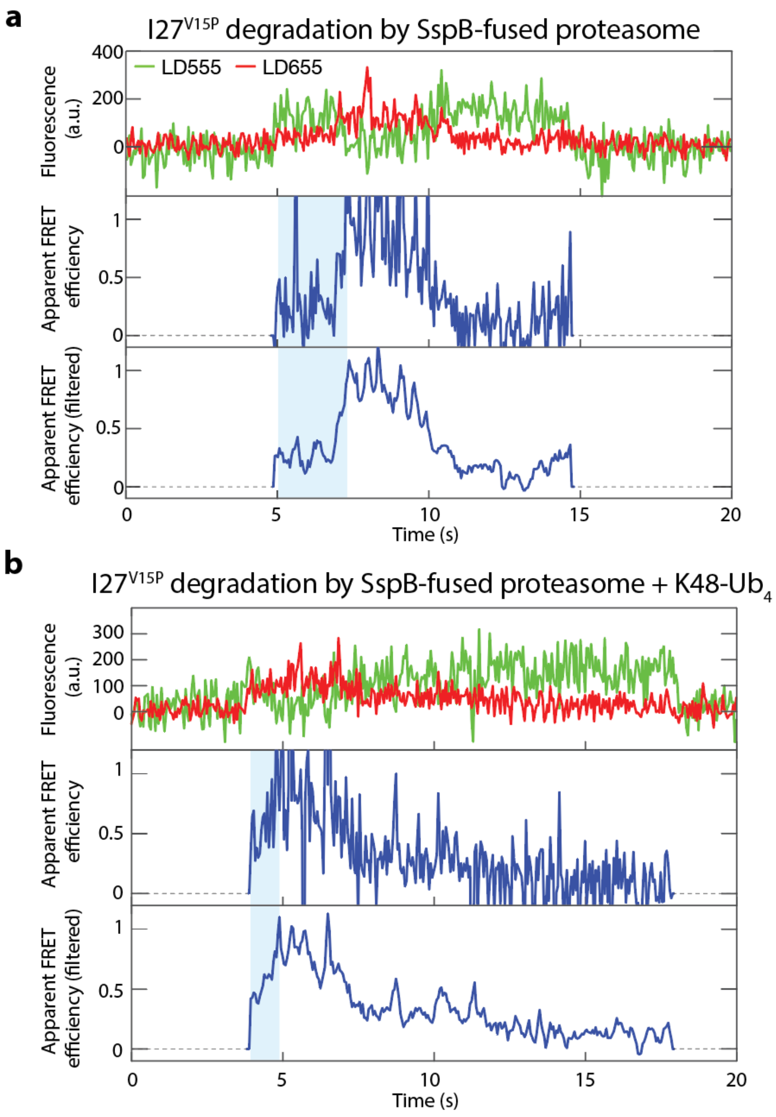

Figure S17: Representative traces for the substrate-processing assay in the absence and presence of ubiquitin chains. Example traces for the SspB-mediated degradation of donor-labeled I27^{V15P} substrate by SspB-fused, acceptor-labeled proteasome **a)** in the absence of ubiquitin chains and **b)** in the presence of K48-linked tetra-ubiquitin chains (K48-Ub_4). Raw donor (LD555, green) and acceptor (LD655, red) fluorescence traces monitored after 532-nm laser excitation, the raw apparent FRET efficiencies, and the filtered apparent FRET efficiencies are shown in the top, middle, and bottom panels, respectively. FRET efficiencies outside of degradation events are not defined and represented by dashed lines. The blue shading indicates the phase of FRET-efficiency increase during substrate-tail insertion, which is accelerated in the presence of ubiquitin chains.

Supplementary Tables:

Substrate	Mean ± s.e.m. (s)	N
Ub-I27^{V15P} + WT proteasome	1.8 ± 0.1	80
Ub-I27^{V15P} + Rpn11^{V80A} proteasome	1.7 ± 0.1	74
I27^{V15P} + SspB-fused proteasome	3.4 ± 0.3	77
I27^{V15P} + SspB-fused proteasome + K48-Ub_4	2.2 ± 0.2	102

Table S1: Substrate tail-insertion and engagement kinetics. Listed are the mean values of the time constants for tail insertion and engagement of either ubiquitinated or SspB-delivered I27^{V15P} substrate, in the absence and presence of ubiquitin chains, as determined by the FRET-based tail insertion and processing assay. N values indicate the number of analyzed events.

Substrate / Proteasome variant	Mean ± s.e.m. (s)	N
Ub-I27^{V15P} + WT proteasome	0.74 ± 0.04	98
Ub-I27$^{V13P/V15P}$ + WT proteasome	0.80 ± 0.05	69
Ub-I27^{V15P} + Rpn11^{V80A} proteasome	0.80 ± 0.04	86

Table S2: Substrate translocation phase prior to unfolding. Listed are the mean values of the time constants for substrate translocation after deubiquitination and prior to unfolding, considering a decay of apparent FRET efficiency from 0.82 to 0.35 in the FRET-based substrate processing assay (see Fig. 1 f). N values indicate the number of analyzed events.

Proteasome variant	k_{s1} (s^{-1})	$k_{non\text{-}s1}$ (s^{-1})
WT proteasome	1.2 ± 0.1	4.5 ± 0.1
WT proteasome + K48-Ub_4	0.4 ± 0.1	4.2 ± 0.1
WT proteasome + K63-Ub_4	0.6 ± 0.1	4.9 ± 0.2
WT proteasome + M1-Ub_4	0.5 ± 0.1	5.9 ± 0.2
Rpn10-ΔUIM proteasome	0.9 ± 0.1	2.1 ± 0.1
SspB-fused proteasome	0.8 ± 0.1	4.2 ± 0.1
SspB-fused proteasome + K48-Ub_4	0.4 ± 0.1	3.8 ± 0.1
Rpn5-mutant proteasome	2.5 ± 0.1	4.2 ± 0.1

Table S3: Rates of proteasome conformational switching. Rates for the conformational transitions from the s1 to non-s1 states (k_{s1}) and from non-s1 to s1 states ($k_{non\text{-}s1}$) were determined by Hidden-Markov-modeling of the results from the FRET-based conformational change assay for wild-type and various mutant proteasomes in the absence and presence of unanchored ubiquitin chains.

Substrate	Mean ± s.e.m. (s)	N
$I27^{V13P/V15P}$	7.3 ± 0.2	102
$I27^{V13P/V15P}$ + K48-Ub_4	7.0 ± 0.1	111
$I27^{V15P}$	10.7 ± 0.6	85
$I27^{V15P}$ + K48-Ub_4	9.0 ± 0.3	113
$I27^{WT}$	47 ± 4	67
$I27^{WT}$ + K48-Ub_4	40 ± 4	66

Table S4: Substrate processing dwells. Given are the mean values for the duration of high-FRET processing dwells measured by the FRET-based conformational change assay during the SspB-mediated degradation of I27-substrate variants in the absence and presence of unanchored ubiquitin chains. The N values indicate the number of analyzed events.

Substrate	Mean ± s.e.m.	N
$I27^{V13P/V15P}$	0	102
$I27^{V13P/V15P}$ + K48-Ub_4	0	111
$I27^{V15P}$	0.7 ± 0.2	85
$I27^{V15P}$ + K48-Ub_4	0.8 ± 0.1	113
$I27^{WT}$	11 ± 2	67
$I27^{WT}$ + K48-Ub_4	10.8 ± 0.7	66

Table S5: Average frequency of s1 excursions per substrate processing dwell. Given are the mean values for the number of excursions from high-FRET non-s1 states to the low-FRET s1 state in the FRET-based conformational change assay during individual SspB-mediated degradation events for I27-substrate variants in the absence and presence of unanchored ubiquitin chains. The N values indicate the number of analyzed events.
